# Bisphenol F and Bisphenol S in a Complex Biomembrane: Comparison with Bisphenol A

**DOI:** 10.3390/jox14030068

**Published:** 2024-09-04

**Authors:** José Villalaín

**Affiliations:** Institute of Research, Development, and Innovation in Healthcare Biotechnology (IDiBE), Universidad “Miguel Hernández”, E-03202 Elche, Alicante, Spain; jvillalain@umh.es; Tel.: +34-648891404

**Keywords:** bisphenol, BPA, BPF, BPS, plasma membrane, molecular dynamics

## Abstract

Bisphenols are a group of endocrine-disrupting chemicals used worldwide for the production of plastics and resins. Bisphenol A (BPA), the main bisphenol, exhibits many unwanted effects. BPA has, currently, been replaced with bisphenol F (BPF) and bisphenol S (BPS) in many applications in the hope that these molecules have a lesser effect on metabolism than BPA. Since bisphenols tend to partition into the lipid phase, their place of choice would be the cellular membrane. In this paper, I carried out molecular dynamics simulations to compare the localization and interactions of BPA, BPF, and BPS in a complex membrane. This study suggests that bisphenols tend to be placed at the membrane interface, they have no preferred orientation inside the membrane, they can be in the monomer or aggregated state, and they affect the biophysical properties of the membrane lipids. The properties of bisphenols can be attributed, at least in part, to their membranotropic effects and to the modulation of the biophysical membrane properties. The data support that both BPF and BPS, behaving in the same way in the membrane as BPA and with the same capacity to accumulate in the biological membrane, are not safe alternatives to BPA.

## 1. Introduction

Exogenous chemicals that interfere with hormone metabolism and hormone signaling, as well as induce damage to endocrine glands, are called endocrine-disrupting chemicals, or EDCs [1,2,3]. EDCs not only bind to hormone receptors but also to many other ones, negatively affecting sexual development, fertility, fetal development, insulin production, growth, and other areas, representing a significant concern for animal and human health [1,2,3]. EDCs may affect and disrupt different metabolic pathways, and they have been directly related to disorders of obesity [4,5,6,7]. EDCs have been found in many places, such as in homes, industrial and derived products, pesticides, food and beverage packaging, cookware, cosmetics, toys, and construction materials, and they are produced/extended by rainfall water, industrial wastes, and indoor and outdoor pollution [8,9,10,11,12]. Moreover, many EDCs are resistant to biodegradation over time or degrade to other chemicals, which might also affect different metabolic pathways. Complicating the issue, many EDCs undergo bioaccumulation with harmful consequences for global health. Lamentably, many EDCs have been found in the atmosphere, rivers, seas, and on land [13,14,15]. Although the first evidence of an EDC, diethylstilbestrol, was described many years ago, the number of EDCs has grown remarkably over time (Endocrine Disruption Exchange, https://endocrinedisruption.org/ (accessed on 5 June 2024)). Additionally, many of them are hydrophobic and, therefore, prone to accumulation in adipose tissue, serving as a pool of EDCs for long durations of time [16]. Because of this, they remain in the body for very long periods of time [5].

**Figure 1:**
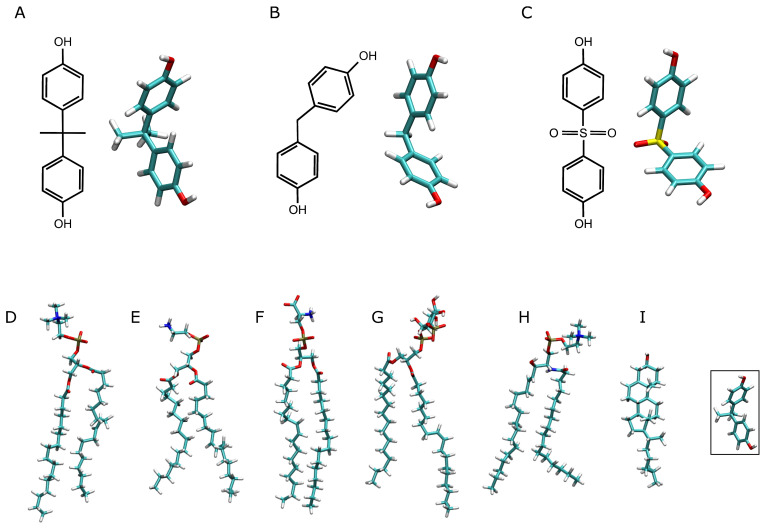
Chemical and molecular structures of (**A**) bisphenol A, (**B**) bisphenol F, and (**C**) bisphenol S, and the molecular structures of the lipid molecules used in this study: (**D**) POPC (1-palmitoyl-2-oleoyl-sn-glycero-3-phosphatidylcholine), (**E**) POPE (1-palmitoyl-2-oleoyl-sn-glycero-3-phosphatidylethanolamine), (**F**) POPS (1-palmitoyl-2-oleoyl-sn-glycero-3-phosphoserine), (**G**) PI-3P (1-palmitoyl-2-oleoyl-sn-glycero-3-phosphoinositol-3-phosphate), (**H**) PSM (N-stearoyl-D-erythro-sphingosylphosphorylcholine) and (**I**) CHOL (cholesterol). The far-right structure depicts the bisphenol A molecular structure in order to compare the relative sizes of the molecules.

Bisphenols are based on two hydroxyphenyl functional groups used for the production of plastic containers, toys, bottles, and medical equipment [5] (Figure 1). Bisphenol A (BPA) (Figure 1A) is used in the manufacture of polycarbonate epoxy resins and plastics and has been shown to exhibit many unwanted effects on animal and human health. BPA is a well-known EDC, and it is produced in the largest quantities worldwide [17]. There are many products in which BPA can be found, such as water bottles, paper, cans, and sealants, and all of them can leak into the environment and food [18,19,20]. It has been shown that BPA affects spermatogenesis and testosterone synthesis, as well as the expression and function of potassium channels; alters membrane fluidity; is diabetogen and obesogen; exhibits developmental toxicity; binds to the human glucocorticoid receptor; and can modulate hormone receptor expression, disrupting the transport of insulin through the membrane and inducing hormone-dependent tumors [21,22,23,24,25,26,27,28,29,30,31,32,33,34,35]. Moreover, BPA affects the uterus and ovarian morphology and function and mammary gland development, and it is linked to cancer [36,37]. BPA has an enormous impact on human health, and its use in daily life must be reconsidered. Over the years, there have been many products that do not use or have reduced their BPA contents, and BPA has been replaced in many applications with other molecules such as bisphenol F (BPF) (Figure 1B) and bisphenol S (BPS) (Figure 1C), as it was considered that these molecules would have a lesser effect on metabolism disruption. However, it seems that this is not the case, since both BPF and BPS, being chemically and structurally similar to BPA, have similar effects as EDCs, possibly sharing the same targets [5,22,29,30,38,39,40,41,42]. These substitutes were selected primarily for their structural similarity, as well as their stability, notwithstanding a poor toxicological evaluation [38,43,44,45]. At the same time, if BPA contents in the human body have, in general, decreased, the presence of its substitutes has increased notably [46,47]. Notably, bisphenols have been found in relatively larger concentrations in obese adults compared to non-obese ones [17,48,49]. Several studies connect adipogenesis and bisphenols in general and BPA in particular [22,50,51,52,53]. Moreover, bisphenols are an important risk factor for diabetes, with β-cells being their primary target [5,40]. As noted, EDCs tend to be lipophilic molecules, and BPA, BPF, and BPS follow this same trend. The xLogP of BPA is 3.3, of BPF is 2.9, and of BPS is 1.9 (PubChem [54]), i.e., all of them have positive values and, therefore, all of them tend to partition into the lipid phase more than into the aqueous one. However, the differences in xLogPs among those molecules tell us that BPA should be the most hydrophobic, whereas BPS should be the least. Although bisphenols tend to be excreted in 24–48 h, prolonged exposure means that, over time, their levels in the body can be relatively high in different organs, tissues, and cells [5].

Molecular dynamics (MD) is perfectly suited to learning about the localization, dynamics, interaction, and structure of bioactive molecules interacting with biomembranes [55,56]. MD simulations have shown that BPA is able to enter a simple membrane in the aqueous phase and tends to form clusters [57]. However, no comparisons have been made between BPA, BPF, and BPS so far. I used MD to define and compare the localization and orientation of BPA, BPF, and BPS in a complex membrane, looking for any interactions with membrane lipids. In doing so, I studied four different systems for each bisphenol molecule (BPA, BPF, and BPS), i.e., twelve systems in total (Table 1). The results I obtained suggest that bisphenols tend to mainly be placed at the membrane interface and, depending on the concentration, can be in the monomer or aggregated state; they form hydrogen bonds with all lipids, except CHOL, and modulate the biophysical properties of the membrane lipids. It is true that I have not performed any biological experiments, since this study only includes molecular dynamics simulations; however, the data obtained in this work support that BPF and BPS act at the same level and with the same capacity to accumulate in biological membranes as BPA.

## 2. Materials and Methods

Unrestrained all-atom MD simulations were carried out using NAMD 3.0b5 [58] and the CHARMM36 lipid force fields [59,60,61]. All the MD parameters used in this work have been described [62,63]. The whole systems were equilibrated before simulation for 10 ns after 100,000 steps of minimization. The production trajectories for each one of the 12 biomembrane systems were obtained for a total of 1000 ns (Table 1). Simulations were carried out at 37 °C and neutral pH.

I have studied four different systems for each one of BPA, BPF, and BPS using a model biomembrane system alike the plasma membrane [64,65,66] (Table 1). Each one of the four systems contained different concentrations of bisphenol molecules as well as different locations, both outside and inside the membrane. The bisphenol molecule is a small one; it is hydrophobic and can associate to form aggregates. I have chosen these systems, containing different amounts of bisphenol to test their ability to insert or remain in the membrane and their association capacity. The concentrations of bisphenols in the membrane do not represent physiological levels. As commented above, my intention has been to compare three types of bisphenol molecules in the membrane under different conditions. All the membrane systems were obtained using the Charmm-Gui web server (http://www.charmm-gui.org (accessed on 4 December 2023) [67]). The systems contained excess water [68] (Table 1). The systems were composed of bisphenol molecules, a membrane, water, and NaCl at physiological conditions, i.e., a concentration of 0.15 M, enclosed in a rectangular box and a neutral setting (Table 1) [69,70,71]. The membranes were composed of approximately 30% of 1-palmitoyl-2-oleoyl-sn-glycero-3-phosphocholine (POPC), 17% of 1-palmitoyl-2-oleoyl-sn-glycero-3-phosphoethanolamine (POPE), 7% of 1-palmitoyl-2-oleoyl-sn-glycero-3-phosphoserine (POPS), 6% of 1-palmitoyl-2-oleoyl-sn-glycero-3-phosphoinositol-3-phosphate (PI-3P), 10% of N-stearoyl-D-erythro-sphingosylphosphorylcholine (PSM), and 30% of cholesterol (CHOL). The exact amount of lipids in each of the systems studied is displayed in Table 1. Additionally, the systems BPA_8, BPF_8, and BPS_8 contained 8 molecules of bisphenols; systems BPA_50, BPF_50, and BPS_50 contained 50 molecules of bisphenols; systems BPA_44, BPF_44, and BPS_44 contained 44 molecules of bisphenols; and systems BPA_45, BPF_45, and BPS_45 contained 45 molecules of bisphenols (Table 1). The chemical structures of the lipid molecules are shown in Figure 1. The presence of one oleoyl chain in the phospholipids increases its fluidity in the membrane and makes the membrane alike the real one. The bilayer normal was parallel to the *z*-axis of the membrane and its surface constituted the x/y plane. The height of the simulation box and the cross-sectional area were permitted to fluctuate independently of each other with no constraints. The bisphenol molecules were created and minimized using Discovery Studio 4.0 (Accelrys Inc., San Diego, CA, USA). The CHARMM General Force Field (CGenFF) compatible stream files of BPA, BPF, and BPS were obtained using the Charmm-Gui web server [67]. The initial arrangements at t = 0 ns are depicted in Supplementary Figures S1–S3 for all the different systems. The systems BPA_8, BPF_8, and BPS_8 contained eight molecules of BP at the external part of the membrane, four on each side of it (Supplementary Figures S1A, S2A and S3A). The systems BPA_50, BPF_50, and BPS_50 contained 50 molecules of BP at the external part of the membrane, 25 on each side of it (Supplementary Figures S1B, S2B and S3B). The systems BPA_44, BPF_44, and BPS_44 contained 44 molecules of BP placed in the middle part of it (Supplementary Figures S1C, S2C and S3C). The systems BPA_45, BPF_45, and BPS_45 contained 45 molecules of BP at the internal part of the membrane, forming a continuous structure in contact with the two external parts of the membrane (Supplementary Figures S1D, S2D and S3D).

The average layer of the lipid phosphate atoms defined the membrane surface and was parallel to the x/y plane. The VMD software was employed for analysis and visualization [62,63,72,73,74]. For the calculation of contacts between molecules, xyz distances were taken into account. The z-distance center-of-masses (COM) and dihedral angles were obtained using standard VMD plugins. S_CD_ order parameters, surface area per lipid, membrane thickness, and molecular areas were calculated as described [74] using VMD “Membplugin” [73]. Mass density profiles were obtained using the VMD “Density Profile Tool” plugin [75]. Hydrogen bonds were defined by a distance less than 3 Å between acceptor and donor atoms and an acceptor-H-donor angle of at least 150° [76]. Unless otherwise stated, the complete simulations were used for analysis, i.e., 1000 ns.

## 3. Results

It has been previously shown that BPA, in the presence of a simple membrane composed of one phosphatidylcholine type, is capable of forming aggregates as well as pores [57]. Taking into account that BPA can be inserted into the membrane, it forms aggregates and is capable of forming a cluster of molecules inside the membrane, 12 different membrane/bisphenol systems have been used (Supplementary Figures S1–S3). These systems have been specifically chosen trying to have different concentrations and arrangements, in order to be able to observe and compare the bisphenols in various situations. 

The time variation in membrane thickness and the time variation in the lipid areas were applied to assess membrane equilibration [77,78]. The mean membrane thickness for all the systems and for the last 40 ns was between 44 and 47 Å as shown in Supplementary Figure S4 and Supplementary Table S1 (rounded to the first decimal). These values are comparable to CHOL-containing systems [79]. Lipid areas remained constant after ~45 ns for all systems except the systems BPA_45, BPF_45, and BPS_45 (Supplementary Figure S5, panels A–C, E–G, and I–K). The average area of all the lipids in the 12 systems for the last 40 ns of MD are shown in Supplementary Table S1, being comparable to those previously reported [74,79,80]. For the systems BPA_45, BPF_45, and BPS_45, a large variation was observed, especially at the beginning of the simulation. This variation decreased over time, but it remained much higher than in the other systems (Supplementary Figure S5, panels D, H, and L). These data indicate that all the systems, except the systems BPA_45, BPF_45, and BPS_45, were equilibrated very early and revealed that the membrane systems reached a steady state after ~45 ns of MD. Lipid areas for the systems BPA_45, BPF_45, and BPS_45, whose membrane thickness was similar to the other ones, were equilibrated later but had a larger fluctuation until the end of the simulation. This difference in lipid areas must be due to the number and disposition of the bisphenol molecules at the beginning of the MD (Supplementary Figures S1–S3, panels D).

I have obtained the variation upon time of the *z*-axis center-of-mass (zCOM) of all the BPx molecules in the membrane compared with the zCOMs of the phosphate atoms at both leaflets, i.e., the membrane surface (Supplementary Figures S6–S9). 

**Figure 2:**
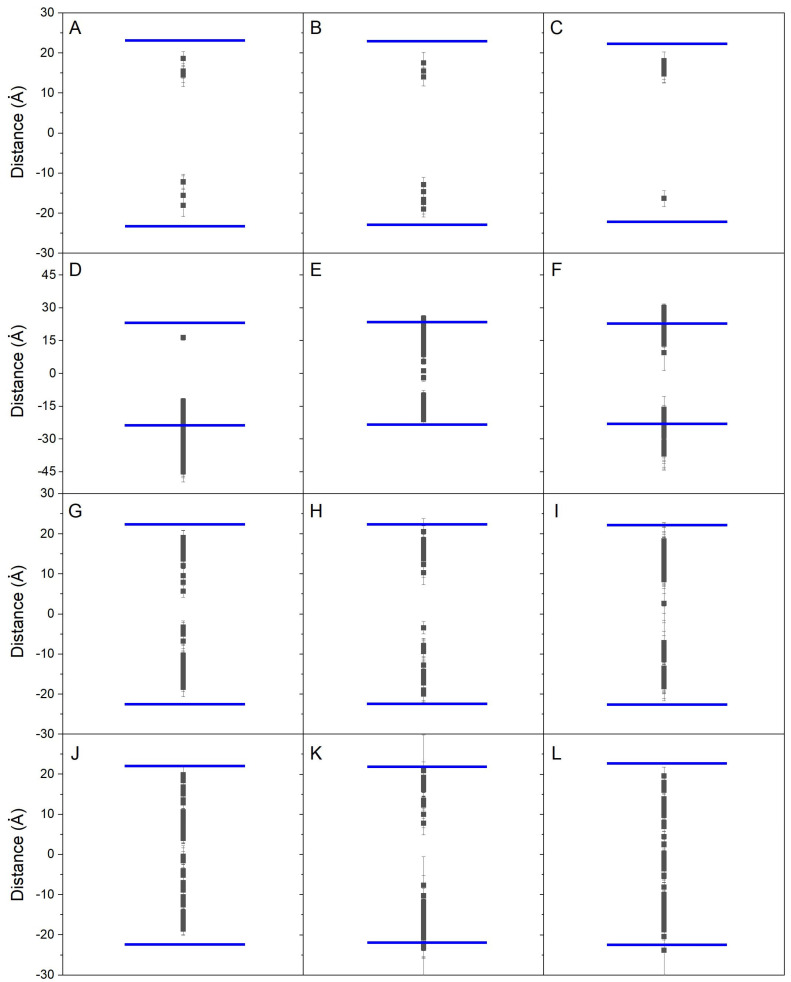
Average zCOM *z*-axis for the last 40 ns of simulation for all the bisphenol molecules in the different systems: (**A**) system BPA_8, (**B**) system BPF_8, (**C**) system BPS_8, (**D**) system BPA_50, (**E**) system BPF_50, (**F**) system BPS_50, (**G**) system BPA-44, (**H**) system BPF_44, (**I**) system BPS_44, (**J**) system BPA_45, (**K**) system BPF_45, and (**L**) system BPS_45. The blue lines depict the average zCOM of the phosphate atoms of the phospholipids, i.e., the membrane surface (middle of the membrane as a reference).

For the systems BPA_8, BPF_8, and BPS_8 (Table 1), 8 molecules of BPx were localized in the water layer at the beginning of the simulation, i.e., outside of the membrane (zCOM data are shown in Supplementary Figure S6A for BPA, Figure S6B for BPF, and Figure S6C for BPS). As seen in the figures, a great variation in the displacement in the zCOM of each one of the molecules in the aqueous solvent was observed for the very first hundreds of ns. However, some of them, and at different times, interacted with the membrane surface, and all those who at some time interacted with the membrane inserted spontaneously into it. In the case of BPA, one BPA molecule contacted the membrane surface at about 4 ns whereas the last one to contact it did so at about 147 ns. In the case of BPF, one BPF molecule contacted the membrane surface at about 2 ns whereas the last one to contact it did so at about 142 ns. In the case of BPS, one BPS molecule contacted the membrane surface at about 8 ns whereas the last one to contact it did so at about 82 ns. Independently of the contact time of each molecule, all of those inserted into the membrane located in the same relative zCOM after a few ns upon incorporation (Supplementary Figure S6). For BPA, the average time since their interaction with the bilayer surface and its insertion into the final zCOM position was 14.9 ± 5.7 ns; for BPF it was 13.5 ± 7.7 ns; and for BPS it was 13.0 ± 6.6 ns. Therefore, the time that elapses between the molecular contact with the surface, its insertion into the membrane, and its final location is quite fast and is also practically identical for all three types of molecules. The average location for the last 40 ns, i.e., the final location, of the BPx molecules with respect to the membrane surface can be observed in Figure 2A (BPA), Figure 2B (BPF), and Figure 2C (BPS). As viewed in the different panels, their location is very similar for all of them and none penetrates beyond the said location; bisphenol molecules are located near its lipid interface, and there are no differences between BPA, BPF, and BPS.

For the systems BPA_50, BPF_50, and BPS_50 (Table 1), 50 molecules of BPx were localized in the water layer of the systems at the start of the simulation, i.e., outside the membrane (zCOM data are shown in Supplementary Figure S7A for BPA, Figure S7B for BPF, and Figure S7C for BPS). Similarly to what was observed in the previous systems, and as shown in the figures, a large variation in the displacement in the zCOM of each one of the molecules in the aqueous solvent was observed, in many cases for the whole simulation time. Nevertheless, some of them, and at different times, interacted with the membrane surface, and all those who at some time interacted with the membrane spontaneously inserted into it. However, and in contraposition to the systems commented previously, the number of bisphenol molecules was considerably larger than in the previous case (from a total number of 50 in this case compared to 8 in the previous one). There was a large fluctuation in the zCOM values for all the bisphenol molecules, but the preponderance of their location was toward the membrane interface. The average location for the last 40 ns, i.e., the final location, of the bisphenol molecules surface can be observed in Figure 2D (BPA), Figure 2E (BPF), and Figure 2F (BPS). As viewed in the different panels, their location is not similar and although there are a few molecules that enter into the membrane, in general, bisphenol molecules tend to be located at the membrane interface. For this situation, nearly all the BPS molecules were inside the membrane but BPA and BPF molecules tend to be both inside and at the surface of the membrane. Again, the differences encountered with the previous situation lie in the number and disposition of the molecules at the beginning of the simulation. As observed in Supplementary Figures S1B, S2B and S3B, many of the molecules, without distinction between BPA, BPF, or BPS, formed dimers, trimers, tetramers, and high-order oligomers, i.e., interacted among them, and aggregated over time. This is the reason why some bisphenol molecules do not insert into the membrane.

For the systems BPA_44, BPF_44, and BPS_44 (Table 1), at the beginning of the simulation, 44 molecules of bisphenol were localized at the middle of the membrane (zCOM data are shown in Supplementary Figure S8A for BPA, Figure S8B for BPF, and Figure S8C for BPS). In this case, and in contrast to the previous systems, where the bisphenol molecules were located in the aqueous solvent, no large fluctuations in zCOM distances over time were observed, except for a small number of molecules. These molecules moved from inside the membrane to the aqueous solvent, but, at some time, interacted again with the membrane surface so that at the end of the simulation, all of them remained inside the membrane. The average location for the last 40 ns, i.e., the final location, of the bisphenol molecules can be observed in Figure 2G (BPA), Figure 2H (BPF), and Figure 2I (BPS). As viewed in the different panels, their location is very similar for all of them and none penetrates beyond said location; thus, bisphenol molecules are located near its lipid interface, and there are no differences between BPA, BPF, and BPS. The molecules are not located in the middle of the membrane, and they have the propensity to be placed near the interfacial part of it. In contrast to the systems BPA_8, BPF_8, and BPS_8, in which there were only 8 molecules in total, in these systems I have 44 molecules, which in some way determines their final location because of the large number of them with respect to the available volume of the hydrophobic part of the membranes for each of the systems studied. As observed in Supplementary Figures S1C, S2C and S3C, and in contrast to the bisphenol molecules in the systems BPA_50, BPF_50, and BPS_50, only a few bisphenol molecules formed low-order oligomers, presumably because they were previously inserted in the middle of the bilayer at the beginning of the MD.

For the systems BPA_45, BPF_45, and BPS_45 (Table 1), 45 molecules of BPx were located inside the membrane but formed an oligomer which expanded the membrane interior and exposed to both membrane surfaces (the zCOM data are shown in Supplementary Figure S9A for BPA, Figure S9B for BPF and Figure S9C for BPS). In this case, and similarly to what was observed in the previous systems, where the bisphenol molecules were located inside the membrane, no large fluctuations in zCOM distances over time were observed, except for a limited number of molecules. These molecules, which sometimes moved from the inside of the membrane to the aqueous solvent, interacted again with the membrane surface, remaining inside the membrane. However, because of the initial disposition of the BP molecules at the beginning, many of them remained close together forming dimers, trimers, or tetramers as well as high-order oligomers. The average location for the last 40 ns, i.e., the final location, of the bisphenol molecules can be observed in Figure 2J (BPA), Figure 2K (BPF), and Figure 2L (BPS). As viewed in the different panels, their final averaged location is very similar and tends to be inside the membrane. Correspondingly to the systems BPA_44, BPF_44, and BPS_44, in which there were 44 molecules, the systems BPA_45, BPF_45, and BPS_45 have 45 molecules, which in some way determine their final location because of the large number of them with respect to the available volume of the hydrophobic part of the membranes for each of the systems studied.

As stated above, the systems BPx_44, BPx_45, and BPx_50 showed the presence of oligomers at different distances, both inside and on the surface of the membrane, which could be the origin of some of the differences mentioned above. Since only the systems BPA_8, BPF_8, and BPS_8 presented bisphenol molecules which did not aggregate over time because of their arrangement in the systems, and they were the ones where all the bisphenol molecules inserted spontaneously into the membrane, I have obtained the absolute average location of all of them in each one of the systems (data obtained from Figure 2A (BPA), Figure 2B (BPF), and Figure 2C (BPS)). In the case of system BPA_8, i.e., BPA, the average position of the molecules with respect to the membrane surface, was 8.0 Å ± 2.3 Å; for system BPF_8, i.e., BPF, was 6.9 Å ± 2.1 Å; and for system BPS_8, i.e., BPS, was 6.2 Å ± 1.2 Å. Therefore, all the bisphenol molecules in the monomer state, i.e., BPA, BPF, and BPS, tend to be located at a similar location inside the bilayer, with an average distance to the membrane surface between 6 and 8 Å.

**Figure 3:**
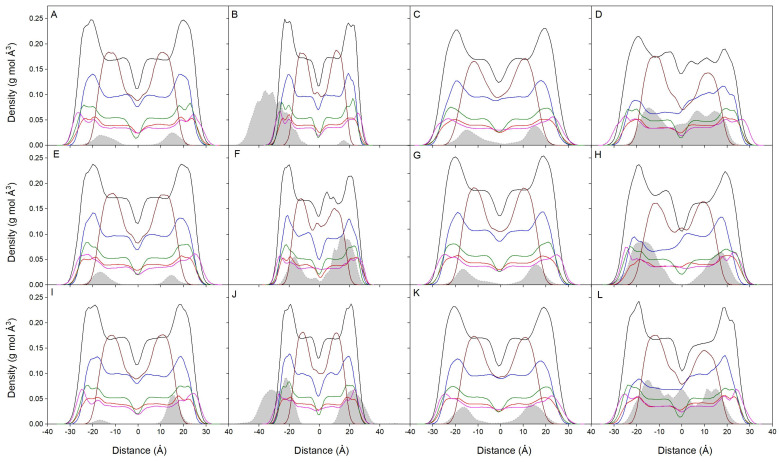
Mass density profiles for the last 40 ns of the MD simulation for (**A**) system BPA_8, (**B**) system BPA_50, (**C**) system BPA_44, (**D**) system BPA_45, (**E**) system BPF_8, (**F**) system BPF_50, (**G**) system BPF_44, (**H**) system BPF_45, (**I**) system BPS_8, (**J**) system BPS_50, (**K**) system BPS_44, and (**L**) system BPS_45. The lipid mass density profiles correspond to POPC (black), POPE (blue), POPS (red), PI-3P (magenta), PSM (green), and CHOL (wine). The bisphenol molecules (BPA in 1–4, BPF in 5–8, and BPS in 9–12) are depicted as gray-shaded dotted black lines. Water and ion mass density profiles have been removed for clarity.

Considering the absolute membrane surface, the favored location of the bisphenol molecules is the one where its zCOM is about 6–8 Å underneath. However, it should be taken into account the real volume the lipids and the bisphenol molecules occupy. The molecule is approximately a cylinder with a radius of about 3Å and a height of about 10Å, so the molecule tends to be positioned in the membrane, a part of it extending about 4–5 Å toward the outside of the membrane and about 4–5 Å toward the inside of it. The mean mass density of all the components for the last 40 ns for all systems is presented in Figure 3. For the systems BPA_8, BPF_8, and BPS_8 (Figure 3A,E,I), the global mass density for all of them is a very similar one between the lipid interface and the oxygen atoms of CHOL. Its location is very well defined; they do not span beyond those limits and there are no differences between BPA, BPF, and BPS. All the lipid profiles are completely symmetric between the two leaflets of the membrane, which implies an equivalent behavior for all the lipids inside the membrane (8 bisphenol molecules compared to 200 lipids in the systems). Figure 3B,F,J show the mass density profiles for the systems BPA_50, BPF_50, and BPS_50, respectively. In this case, 50 molecules of bisphenol, as opposed to 8 in the previous systems, were in the water layer of the systems at the beginning of the simulation. As observed in the figures, the bisphenol molecules tend to insert into the membrane, near the lipid interface. However, it is also possible to see that there is a grouping of bisphenol molecules (Supplementary Figures S1B, S2B and S3B) forming different order oligomers, which presumably prevent many of them from inserting into the membrane unless they were in the form of monomers (see the small band in Figure 3B). The lipid profiles tend to be symmetric between the two layers, but there are some small differences due to the large number of bisphenol molecules in the membrane (50 bisphenol molecules compared to 200 lipids in the systems). The mass density profiles for the systems BPA_44, BPF_44, and BPS_44 are shown in Figure 3C,G,K, respectively. In this case, 44 molecules of bisphenol were localized at the beginning of the simulation in the middle part of the membrane without touching each other, i.e., in the monomer form (Supplementary Figures S1C, S2C and S3C). The bisphenol molecules, regardless of whether they can form some oligomers along the simulation, at the end of it are located at the lipid interface. All the lipid profiles are almost symmetric between the two layers, which would imply a similar behavior for all of them (44 bisphenol molecules compared to 200 lipids in the systems). Figure 3D,H,L show the mass density profiles for the systems BPA_45, BPF_45, and BPS_45, respectively. In this case, 45 molecules of bisphenol were located in the membrane forming a high-order aggregate which completely spanned the width of the membrane (Supplementary Figures S1D, S2D and S3D). Throughout the simulation the bisphenol molecules tend to expand through the membrane interior; although, depending on concentration, they tend to oligomerize and the spreading apart takes some time. This is more notorious for the systems BPA_45 and BPS_45, i.e., BPA and BPS, where the bisphenol molecules expand the interior of the membrane, and continue to form oligomers but tend to flee from the surface (Figure 3D,L, respectively). For BPF, although they form oligomers, they spread apart from the middle of the membrane and localize at the interface (Figure 3H). Because of the high number of bisphenol molecules and the formation of high-order oligomers, the lipid profiles do not tend to be symmetric between the two membrane layers, there being some differences between leaflets depending on the location of the oligomers (45 bisphenol molecules compared to 200 lipids in the systems). It can be observed that all the bisphenol molecules, whether in the monomeric or aggregated form, tend to insert in the membrane and locate at its interface, with no differences between BPA, BPF, or BPS.

**Figure 4:**
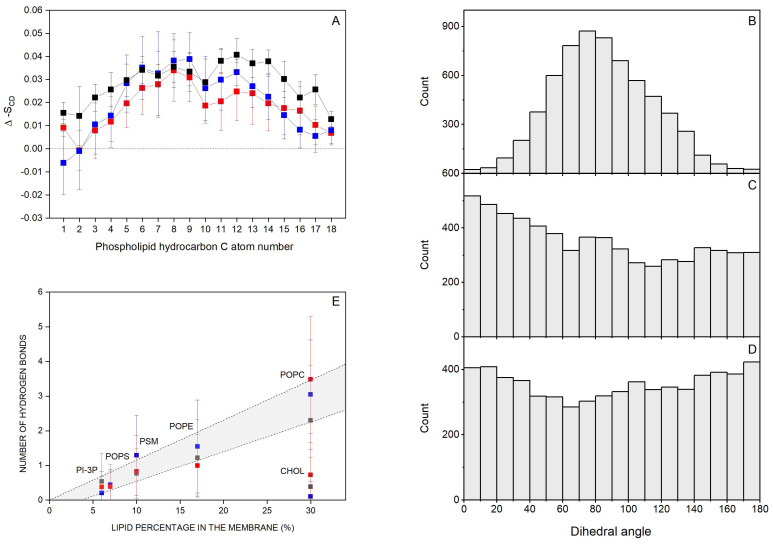
(**A**) Average deuterium order parameter, S_CD_, difference between the bulk phospholipids and phospholipids within 5 Å of bisphenol molecules for system BPA_8 (-■-), system BPF_8 (-■-), and system BPS_8 (-■-); dihedral angles for (**B**) BPA (system BPA_8), (**C**) BPF (system BPF_8), and (**D**) BPS (system BPS_8); and (**E**) average number of hydrogen bonds between the lipids and BPA (system BPA_8, -■-), BPF (system BPF_8, -■-), and BPS (system BPS_8, -■-).

Molecules which interact with membranes can disturb the hydrocarbon chain order, i.e., the deuterium order parameter S_CD_ [81]. Thus, I have considered the effect of the bisphenol molecules on the S_CD_ of the *sn*-1 and *sn*-2 acyl chains of the phospholipids. Since only for the systems BPA_8, BPF_8, and BPS_8 the bisphenol molecules remain completely in the monomer form for all the simulation time and there is no aggregation at all, I have measured the S_CD_ parameter for these systems (Supplementary Figures S10, S11 and S12 for the systems BPA_8, BPF_8, and BPS_8, respectively). For system BPA_8, i.e., the system containing BPA, the average S_CD_ values of the bulk phospholipids, i.e., POPC, POPE, POPS, PI-3P, and PSM, were in accordance with the profiles observed earlier [61,82,83] (Supplementary Figure S10). For the same phospholipids near the BPA molecules, there were significant changes in the S_CD_ profiles since a decrease in the S_CD_ values was observed. The decrease in S_CD_ observed for the phospholipids would indicate that BPA would augment the fluidity of the hydrocarbon chains of all the phospholipids in the membrane. For the systems BPF_8 and BPS_8, i.e., the systems containing BPF and BPS, respectively, the average S_CD_ values of the acyl chains of the bulk phospholipids agreed with the profiles previously observed [61,82,83] as was the case for BPA (Supplementary Figures S11 and S12, respectively). The effect of BPF and BPS on the S_CD_ values for phospholipids near the bisphenol molecules was, again, significant, since a decrease in the S_CD_ values was observed. As before, the decrease in S_CD_ would indicate that both BPF and BPS augment the fluidity of the hydrocarbon chains of all the phospholipids in the membrane. From these data, it can be inferred that the bisphenol molecules insert in between the hydrocarbon chains of the phospholipids, and they do not show a dramatic effect on their anisotropy but augment the fluidity of the membrane. I have measured the difference between the S_CD_ values pertaining to the bulk phospholipids and the phospholipids within 5 Å of BPA, BPF, and BPS molecule-containing systems and calculated for all the carbon atoms of the hydrocarbon chains of all the phospholipids in order to check whether there were any significant differences between them (Figure 4A). As observed in Figure 4A, the differences are very similar for all the phospholipids in the membrane and for all the carbon atoms of the hydrocarbon chains in the vicinity of the different bisphenols. The effect of the bisphenol molecules on the fluidity of the phospholipids is very similar, with no significant differences between them. This would imply that the insertion and interaction of the three molecules, BPA, BPF, and BPS, in the hydrocarbon chains are similar.

I have obtained the dihedral angle between the hydroxyphenyl moieties of the different bisphenols and the results are shown in Figure 4B (BPA, system BPA_8), Figure 4C (BPF, system BPF_8), and BPS (Figure 4D, system BPS_8). As can be seen in the figures, there is a large distribution in the angles, especially in the systems containing BPF and BPS, since they cover all the angles from 0° to 180° and have no preference at all for anyone (Figure 4C,D, respectively). BPA in the system BPA_8 also shows a large dispersion but is relatively centered at about 80° ± 40° (Figure 4B). These data could mean that the presence of the two methyl groups in the middle of the BPA molecule can limit its conformation, while it would be more flexible for both BPF and BPS.

I have determined the mean number of hydrogen bonds between the bisphenol molecules and each of the lipids for systems BPA_8 (BPA), BPF_8 (BPF), and BPS_8 (BPS), and the results are shown in Figure 4E. There is a direct relationship between the number of hydrogen bonds between the bisphenol molecules and the relative abundance of the lipids in the membrane, except for CHOL. In this case, CHOL is as abundant as POPC, but the number of hydrogen bonds between the bisphenol molecules and CHOL is about three times less than the hydrogen bonds between the bisphenols and POPC. It would be possible that the specific location of bisphenols in the membrane near the phospholipid interface rather than near the oxygen atom of CHOL would be the cause of this difference. Nevertheless, these data would show that the bisphenol molecules are less likely to bind the CHOL molecules in the membrane and all three bisphenol molecules would have the same tendency to interact with phospholipids with no significant differences between the three of them.

For the systems BPA-8, BPA_50, BPF-8, BPF_50, BPS-8, and BPS_50, the bisphenol molecules were placed in both water layers, and for the systems BPA_44, BPA_45, BPF_44, BPF_45, BPS_44, and BPS_45, in the middle of the membrane (Supplementary Figures S1–S3). The bisphenol molecules, which at the beginning of the simulation were located in the solvent, moved to a position near the membrane interface, whereas the bisphenol molecules, which at the beginning of the simulation were inserted in the middle of the membrane, moved to a place close the membrane interface. However, many of the bisphenol molecules, depending on the different systems, aggregated over time. Not only did they form dimers, trimers, or tetramers but also high-order oligomers (Table 2). For the systems BPA_8, BPF_8, and BPS_8, all the bisphenol molecules were spontaneously inserted into the membrane and all of them remained in the monomeric state until the end of the MD (Table 2). For the systems BPA_50, BPF_50, and BPS_50, nearly all the bisphenol molecules were inserted into the membrane and about 90%, i.e., the vast majority, aggregated and formed high-order oligomers until the end of the simulation (Table 2). For the systems BPA_44, BPF_44, and BPS_44, all bisphenols remained inside and a great proportion of them, i.e., about 65% of the total bisphenol molecules, remained in the monomeric state until the end of the simulation (Table 2). For the systems BPA_45, BPF_45, and BPS_45, where 45 bisphenol molecules span their whole width, all of them remained inside it and a great proportion of them, i.e., about 80% of the total bisphenol molecules, remained in the aggregated state until the end of the simulation (Table 2). It is clear that bisphenol molecules aggregate in a concentration-dependent mode and, independently of the way they were located at the beginning of the simulation, they do so inside the membrane if their concentration is above a certain threshold. 

**Figure 5:**
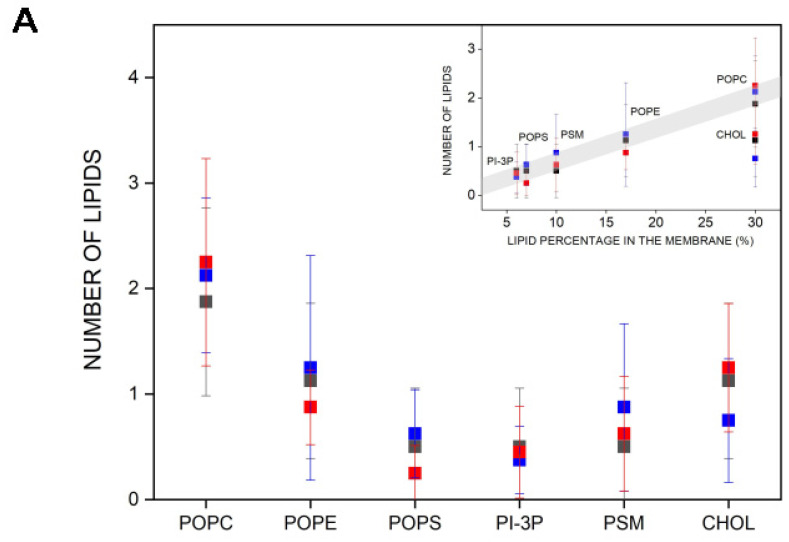

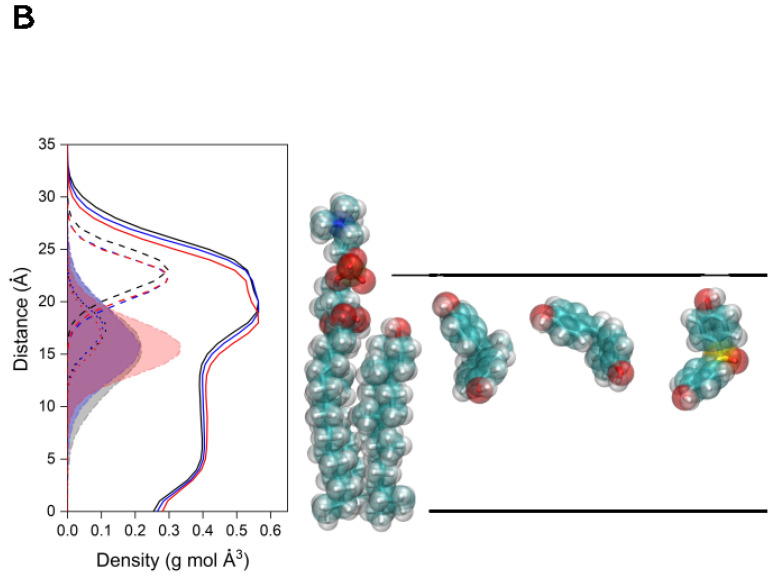
(**A**) Average number of lipid molecules near the bisphenol molecules for the last 40 ns of MD simulation: system BPA_8 (-■-), system BPF_8 (-■-), and system BPS_8 (-■-). The insert represents the number of lipids near the bisphenols relative to the lipid percentage in the membrane. (**B**) Global average mass density profiles for the last 40 ns of the MD simulation for system BPA_8 (black), system BPF_8 (blue), and system BPS_8 (red) for the phospholipids (continuous line); phospholipid phosphates (dashed line); the oxygen atom of CHOL (dotted line); and the bisphenol molecules (shadowed dotted/dashed lines) and a pictorial view of the average relative position of POPC, CHOL, BPA, BPF, and BPS (from left to right).

I have determined the number and type of lipids that are within 5 Å of the bisphenol molecules, i.e., in close contact (Figure 5A). I have measured the data pertaining to systems BPA_8, BPF_8, and BPS_8 because, as commented above, the bisphenol molecules in these systems are always in the monomer state and they do not interact with each other at any moment of the molecular dynamics simulation (see above). The average number of POPC molecules near the bisphenols is about two, and the same for BPA, BPF, and BPS. In the case of POPE and CHOL, the average number is one and for POPS, PI-3P, and PSM, it is less than one (Figure 5A). In all cases, the number of lipids is nearly identical for the three bisphenol molecules, i.e., BPA, BPF, and BPS. By plotting the number of lipid molecules versus the lipid percentage in the membrane, it is possible to see that there is practically a linear relationship between both parameters, i.e., the larger the number of lipids in the membrane, the larger the number of lipids near the bisphenol molecules, with one exception, CHOL (insert, Figure 5A). The number of CHOL molecules near the bisphenol molecules is not related to its quantity in the membrane, since it would have to have a greater number than what is already observed and there are no differences between BPA, BPF, or BPS (insert, Figure 5A). I have also obtained the radial distribution function for the lipid molecules around the membrane-inserted bisphenol molecules for the last 40 ns of simulation (Supplementary Figure S13A for system BPA_8, Figure S13B for system BPF_8, and Figure S13C for system BPS_8, respectively). It can be observed in the figures that all three bisphenol molecules exclude CHOL, in agreement with the fact that the number of hydrogen bonds between the CHOL molecule and the bisphenol molecules is much lower than with other lipids (Figure 4E).

## 4. Discussion

EDCs are exogenous chemicals that interfere with hormone metabolism and signaling, damage endocrine glands, and disrupt many metabolic pathways, posing a significant concern for animal and human health [1,2,3,4,5,6,7]. EDCs are found in the home, industries, as well as in the atmosphere, rivers, sea, and land; are resistant to biodegradation over time; and bioaccumulate through the food chain [5,8,9,10,11,12,13,14,15]. Disappointingly, the number of EDCs has grown remarkably over time and the list grows every year. Furthermore, the vast majority is lipophilic and, therefore, prone to accumulate in the adipose tissue serving as an EDC pool [16]. BPA, a well-known EDC, was the first and main chemical used in the production of plastics and resins but, over time, has been recognized to exhibit many undesirable effects on animal and human health [17,18,19,20,21,22,23,24,25,26,27,28]. BPA has been replaced in many applications by other similar molecules such as BPF and BPS in the consideration that these molecules would have a lesser effect on metabolism disruption. As commented above, these substitutes were selected primarily for their structural similarity despite poor toxicological evaluation [38,43,44,45]. In fact, many studies have shown that BPA, BPF, and BPS display similar biological effects [5,22,29,30,38,39,40,41,42,84,85,86].

I have used MD to describe the localization and orientation of BPA, BPF, and BPS in the membrane and have determined the presence of any interactions with its lipidic components in order to define, if possible, the mechanism of action of the bisphenols at the membrane level as well as whether there are any differences between them. For that goal, I have used 12 membrane/bisphenol systems, differentiated by the number and location of the bisphenol molecules in the systems and trajectories obtained for a total of 1000 ns for each one. The systems were relatively well equilibrated as assessed by measuring the membrane thickness and lipid areas. The systems BPA_8, BPF_8, and BPS_8 consisted of a membrane and eight bisphenol molecules located in the water layer at the beginning of the MD simulation. The bisphenol molecules behaved in the same way, being in the monomeric state all the time and located near the membrane interface without penetrating into the membrane. The systems BPA_50, BPF_50, and BPS_50 consisted of a membrane and 50 bisphenol molecules located in the water layer at the beginning of the simulation. Over time, there was a large oscillation in their zCOM values, but the preponderance of their location was toward the membrane interface. The differences encountered with the previous situation lie in the number and disposition of the molecules at the beginning. In this case, because of the great number of molecules in the systems and without any distinction between the bisphenols, they interacted among themselves and aggregated with time (described previously [57]). This should be the reason why some bisphenol molecules do not insert into the membrane. The systems BPA_44, BPF_44, and BPS_44 consisted of 44 bisphenol molecules and were located at the middle part of the membrane at the beginning of the simulation. In this case, no large fluctuations in the zCOM distances over time were observed, except for a small number of molecules. Again, the bisphenol molecules are located near the lipid interface. However, a few numbers of bisphenol molecules formed low-order oligomers. The systems BPA_45, BPF_45, and BPS_45 consisted of 45 molecules of bisphenol molecules forming a high-order oligomer which expanded the membrane interior and was exposed to both membrane surfaces. Being inserted in the membrane, no large fluctuations in the zCOM distances over time were observed, but the majority remained close together forming different-order oligomers. It can be clearly observed that all bisphenols, whether in monomeric or aggregated form, tend to integrate into the membrane and locate at its interface, with no significant differences between BPA, BPF, or BPS.

The systems BPA_8, BPF_8, and BPS_8 presented bisphenol molecules which did not aggregate over time and they were the ones where all the bisphenol molecules were inserted spontaneously into the membrane. Because of that, I studied these systems in depth. All the bisphenol molecules in these systems, i.e., BPA, BPF, and BPS, were located at a similar position inside the membrane, with a mean distance of the molecule center to the membrane surface between 6 and 8 Å. Therefore, the whole bisphenol molecule, without any distinction between BPA, BPF, and BPS, lies between the phosphate atoms of the phospholipids and the oxygen atom of CHOL. By studying the S_CD_ order parameter of the phospholipids, it is possible to say that the bisphenols increase the fluidity of the hydrocarbon chains of all the phospholipids in the membrane, and insert relatively well in between the hydrocarbon chains of the phospholipids. The effect on the S_CD_ order parameter is very similar for all the bisphenols studied, confirming again that there are no differences among them and they act in the same way. I have obtained the dihedral angle between the hydroxyphenyl moieties of the bisphenols, where BPF and BPS show a large distribution in the angles without any preference. However, BPA has a large dispersion but is relatively centered at about 80° ± 40°. These data mean that both BPF and BPS are more flexible than BPA, perhaps due to the presence of the two methyl groups in the middle of the molecule. I have found that there is a direct relationship between the number of hydrogen bonds among the bisphenol molecules and the relative abundance of the lipids, except CHOL, in the membrane, the specific location of the bisphenols in the membrane being the presumed cause of this difference. Nevertheless, the bisphenol molecules are less likely to bind the CHOL molecules in the membrane and all three bisphenol molecules have the same tendency to interact with phospholipids without any significant differences among the three of them. There is a practically linear relationship between the number of lipids in the membrane and the number of lipids near the bisphenol molecules, except CHOL. These data agree with the fact that the number of hydrogen bonds between the CHOL molecule and the bisphenol ones is much lower than with other lipids [87]. Apart from that, there are no differences between BPA, BPF, and BPS. It is worth noting that many of the bisphenol molecules, depending on the different systems under study, aggregated over time. Not only do they form dimers, trimers, or tetramers but also high-order oligomers in a concentration-dependent mode and, independently of the way they were located at the beginning of the simulation, they can aggregate inside the membrane. It is important to highlight this fact, since, as previously mentioned, bisphenols tend to accumulate over time and there is the possibility that at low concentrations they exist as monomers in the membrane, but at high concentrations, they could form oligomers, and, therefore, have different effects on the cells.

The global average mass density profiles for the last 40 ns for the systems BPA_8, BPF_8, and BPS_8 are shown in Figure 5B. Given that in these systems there are eight molecules in the membrane and no aggregation is observed among them at any time, it could be implicitly considered that there is a need for a statistical analysis of the results obtained for each type of molecule. There are small differences in the average mass densities for the phospholipids in the three systems as well as with their phosphate atoms but the overall membrane structure is similar as expected. Similarly, the average mass densities of the oxygen atoms of CHOL are comparable. Interestingly, the mean localization of the bisphenol molecules is nearly identical for each one of the membrane systems, approximately centered on the CHOL oxygen atom and the phospholipid carbonyl groups (Figure 5B). Although the dispersion showed by BPS is slightly lower than that of both BPA and BPF, their average position is the same (Figure 5B). Accordingly, these results suggest that bisphenols are located at the membrane interface, they have no preferred orientation inside the membrane, depending on concentration can be in the monomer or aggregated state, and they form hydrogen bonds with all lipids except CHOL depending on the lipid concentration and affect the biophysical properties of the membrane lipids. However, I have not observed the formation of any pore for any of the studied systems. One of the most interesting facts is that bisphenol molecules tend to exclude CHOL. 

It is true that I have not performed any biological experiments as this study only includes molecular dynamics simulations. However, the data obtained in this work suggest that bisphenols affect membranes in a similar way because of their membranotropic effects and, consequently, cells through the modulation of membrane biophysical properties. In any case, these data support that the properties of both BPF and BPS are similar to those of BPA [5,22,29,30,38,39,40,41,42,84,85,86]. Therefore, both BPF and BPS, acting at the same level and with the same capacity to accumulate in the biological membrane as BPA, are not a safe alternative to BPA.

## 5. Conclusions

BPA, BPF, and BPS localize at the membrane interface between the phospholipid phosphate atoms and the oxygen atom of CHOL; they have no preferred orientation in the membrane; they can oligomerize; and they form hydrogen bonds with all lipids except CHOL depending on the lipid concentration and affect the biophysical properties of the membrane lipids. Both BPF and BPS, acting at the same level and with the same capability to accumulate in the membrane as BPA, are not a safe alternative to it.

## Figures and Tables

**Table 1:** Systems and number of the molecules/atoms used in this study. The NaCl concentration was 0.15 M. The production trajectories for each one of the 12 different systems were calculated for 1000 ns. BPx refers to either BPA, BPF, or BPS (lipid abbreviations as in the legend in Figure 1).

Systems	BPx_8	BPx-50	BPx_44	BPx_45
BPx	8	50	44	45
POPC	60	60	180	54
POPS	34	34	102	27
POPS	14	14	42	13
PI-3P	12	12	36	12
PSM	20	20	60	18
CHOL	60	60	180	53
LIPIDS	200	200	600	177
H_2_O	9440	8954	41,216	9602
Na^+^	77	75	267	76
Cl^−^	27	25	117	27
Initial dimensions x-y-z (Å)	83-83-100	84-83-99	141-141-123	84-84-100

**Table 2:** Number of oligomer types and number of molecules forming them for the systems studied in this work.

System	BPA_8	BPA_50	BPA_44	BPA_45	BPF_8	BPF_50	BPF_44	BPF_45	BPS_8	BPS_50	BPS_44	BPS_45
Number of Molecules in Oligomer												
1 (monomer)	8	4	30	8	8	4	28	11	8	5	28	11
2	-	-	2	-	-	3	5	4	-	2	4	1
3	-	-	-	3	-	2	2	-	-	-	-	-
4	-	-	1	-	-	1	-	-	-	-	-	-
5	-	-	-	1	-	-	-	2	-	-	-	-
6	-	-	1	-	-	-	-	-	-	1	-	-
7	-	-	-	-	-	-	-	-	-	-	-	-
8	-	-	-	1	-	-	-	-	-	-	1	-
9	-	1	-	-	-	-	-	-	-	-	-	-
>10	-	2	-	2	-	1	-	2	-	2	-	1
Total	8	50	44	45	8	50	44	45	8	50	44	45

## Data Availability

Data can be requested from the author.

## Supplementary Materials

The following supporting information can be downloaded at: https://www.mdpi.com/article/10.3390/jox14030068/s1, Figure S1: Lateral and apical views of the initial, t = 0 ns, and final, t = 1000 ns, disposition of (A) system BPA_8, (B) system BPA_50, (C) system BPA_44, and (D) system BPA_45; Figure S2: Lateral and apical views of the initial, t = 0 ns, and final, t = 1000 ns, disposition of (A) system BPF_8, (B) system BPF_50, (C) system BPF_44, and (D) system BPF_45; Figure S3: Lateral and apical views of the initial, t = 0 ns, and final, t = 1000 ns, disposition of (A) system BPS_8, (B) system BPS_50, (C) system BPS_44, and (D) system BPS_45; Figure S4: Time variation of membrane thickness for the whole simulation time and average histograms for the membrane thickness for the last 40 n of the simulation for (A) system BPA_8, (B) system BPA_50, (C) system BPA_44, (D) system BPA_45, (E) system BPF_8, (F) system BPF_50, (G) system BPF_44, (H) system BPF_45, (I) system BPS_8, (J) system BPS_50, (K) system BPS_44 and (L) system BPS_45; Figure S5: Time variation of lipid areas for the whole simulation time for the last 40 n of the simulation for (A) system BPA_8, (B) system BPA_50, (C) system BPA_44, (D) system BPA_45, (E) system BPF_8, (F) system BPF_50, (G) system BPF_44, (H) system BPF_45, (I) system BPS_8, (J) system BPS_50, (K) system BPS_44 and (L) system BPS_45; Figure S6: Time variation of the z-axis distance (middle of the membrane as a reference) for the different BP molecules in (A) system BPA_8 (8 molecules of BPA), (B) system BPF_8 (8 molecules of BPF) and (C) system BPS_8 (8 molecules of BP); Figure S7: Time variation of the z-axis distance (middle of the membrane as a reference) for the different BP molecules in (A) system BPA_50 (50 molecules of BPA), (B) system BPF-50 (50 molecules of BPF) and (C) system BPS_50 (50 molecules of BP). The z-axis distance of the phosphate atoms of the phospholipids, defining the membrane surface, is depicted in black (upper and lower boundaries); Figure S8: Time variation of the z-axis distance (middle of the membrane as a reference) for the different BP molecules in (A) system BPA_44 (44 molecules of BPA), (B) system BPF_44 (44 molecules of BPF) and (C) system BPS_44 (44 molecules of BP). The z-axis distance of the phosphate atoms of the phospholipids, defining the membrane surface, is depicted in black (upper and lower boundaries); Figure S9: Time variation of the z-axis distance (middle of the membrane as a reference) for the different BP molecules in (A) system BPA_45 (45 molecules of BPA), (B) system BPF_45 (45 molecules of BPF) and (C) system BPS_45 (45 molecules of BP); Figure S10: Average deuterium order parameter –SCD calculated for the hydrocarbon chains of the phospholipids in system BPA_8. (A, C, E, G) oleoyl and (B, D, F, H) palmitoyl acyl chains of (A, B) POPC, (C, D) POPE, (E, F) POPS and (G, H) PI-3P as well as the palmitoyl (I) and sphyngosyl (J) acyl chains of PSM; Figure S11: Average deuterium order parameter –SCD calculated for the hydrocarbon chains of the phospholipids in system BPF_8. (A, C, E, G) oleoyl and (B, D, F, H) palmitoyl acyl chains of (A, B) POPC, (C, D) POPE, (E, F) POPS and (G, H) PI-3P as well as the palmitoyl (I) and sphyngosyl (J) acyl chains of PSM; Figure S12: Average deuterium order parameter –SCD calculated for the hydrocarbon chains of the phospholipids in system BPS_8. (A, C, E, G) oleoyl and (B, D, F, H) palmitoyl acyl chains of (A, B) POPC, (C, D) POPE, (E, F) POPS and (G, H) PI-3P as well as the palmitoyl (I) and sphyngosyl (J) acyl chains of PSM; Figure S13: Radial distribution function, g(r), corresponding to the lipid molecules around the membrane-inserted bisphenol molecules for (A)) system BPA_8, (B) system BPF_8 and (C) system BPS_8; Table S1: Average molecular area (Å2) and membrane thickness (Å) for the last 40 ns of the simulation for all the lipids in the systems studied in this work (mean ± SD).

## Abbreviations

BPx	Bisphenols (BPA, BPF or BPS)
BPA	4-[2-(4-hydroxyphenyl)propan-2-yl]phenol
BPF	4-[(4-hydroxyphenyl)methyl]phenol
BPS	4-(4-hydroxyphenyl)sulfonylphenol
CHOL	Cholesterol
EDC	Endocrine-disrupting chemicals
MD	Molecular dynamics
PI-3P	1-Palmitoyl-2-oleoyl-sn-glycero-3-phosphoinositol-3-phosphate
POPC	1-Palmitoyl-2-oleoyl-sn-glycero-3-phosphocholine
POPE	1-Palmitoyl-2-oleoyl-sn-glycero-3-phosphoethanolamine
POPS	1-Palmitoyl-2-oleoyl-sn-glycero-3-phospho-L-serine
PSM	N-Palmitoyl-D-erythro-sphingosylphosphorylcholine
zCOM	*z*-axis Centre of mass
