# Supplementary material for: Bisphenol F and Bisphenol S in a Complex Biomembrane: Comparison with Bisphenol A
Source: J Xenobiot. 2024 Sep 4;14(3):1201–20. doi: 10.3390/jox14030068 (PMC11417855; doi:10.3390/jox14030068)
Supplement: Supplementary file 1 [file jox-14-00068-s001.zip › jox-3098793-supplementary.pdf]

Journal of
Xenobiotics

MDPI

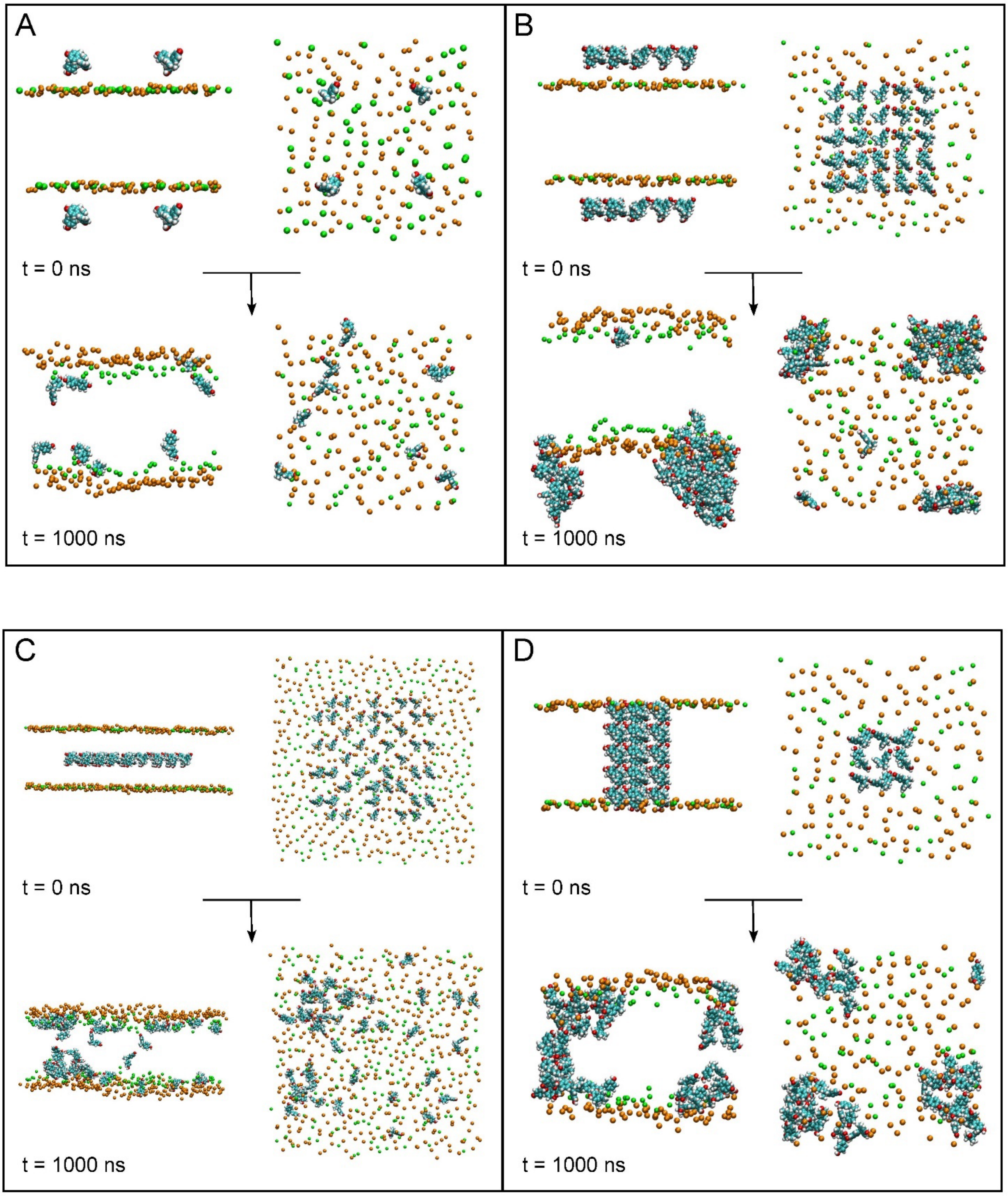

Figure S1. Lateral and apical views of the initial, t=0 ns, and final, t= 1000 ns, disposition of (A) system BPA_8, (B) system BPA_50, (C) system BPA_44 , and (D) system BPA_45 (Table 1). The disposition of the BPA molecules in each one of the systems are also displayed. The BPA molecules are depicted in VDW representation, the phosphate atoms of the phospholipids, defining the upper and lower boundaries of the membrane, are depicted in VDW representation and orange colour and the oxygen atoms of cholesterol are depicted in VDW representation and green colour. The lipid and water molecules and the chloride and sodium ions have been removed for clarity.

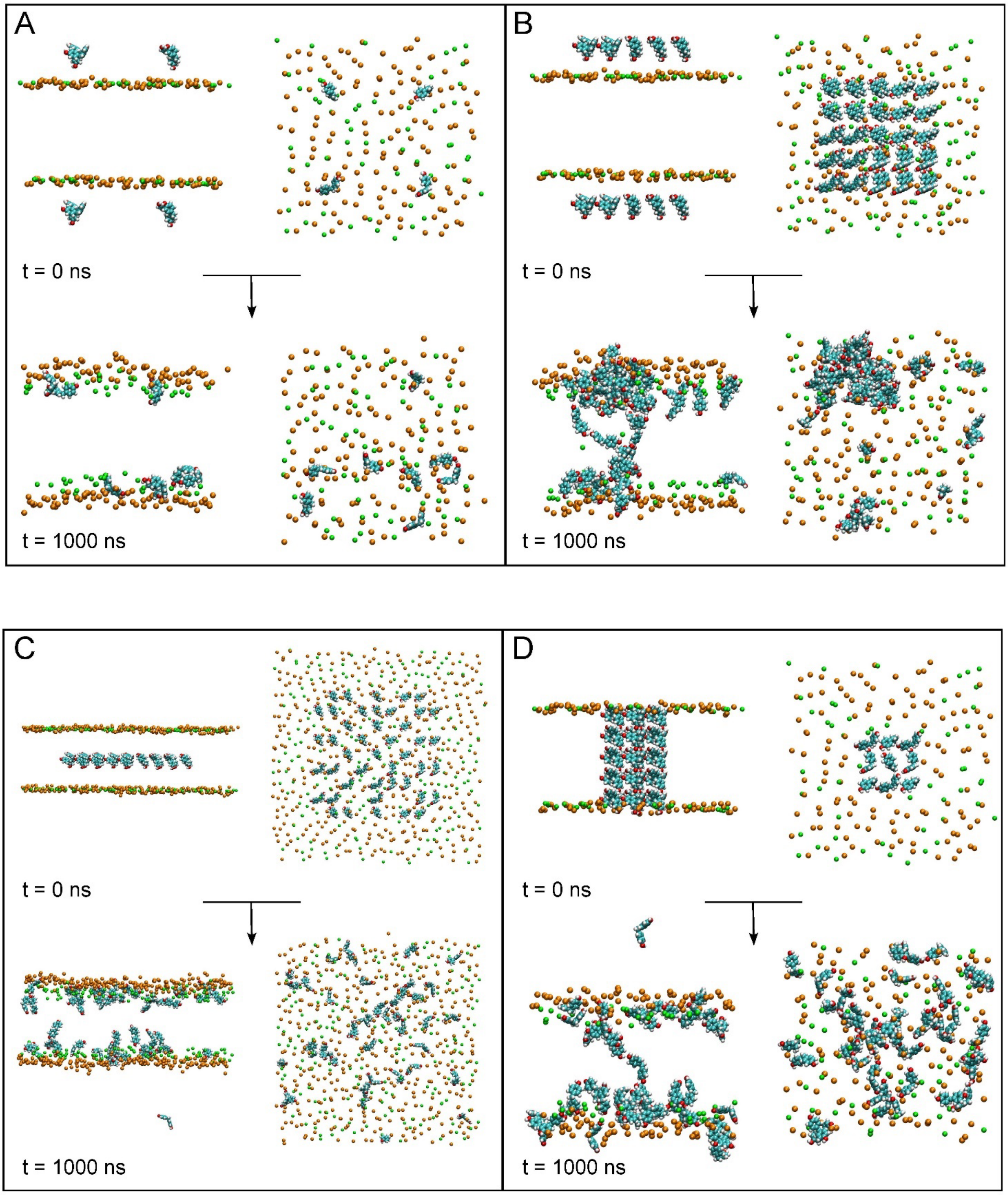

Figure S2. Lateral and apical views of the initial, t=0 ns, and final, t= 1000 ns, disposition of (A) system BPF_8, (B) system BPF_50 , (C) system BPF_44 , and (D) system BPF_45 (Table 1).The disposition of the BPF molecules in each one of the systems are also displayed. The BPF molecules are depicted in VDW representation, the phosphate atoms of the phospholipids, defining the upper and lower boundaries of the membrane, are depicted in VDW representation and orange colour and the oxygen atoms of cholesterol are depicted in VDW representation and green colour. The lipid and water molecules and the chloride and sodium ions have been removed for clarity.

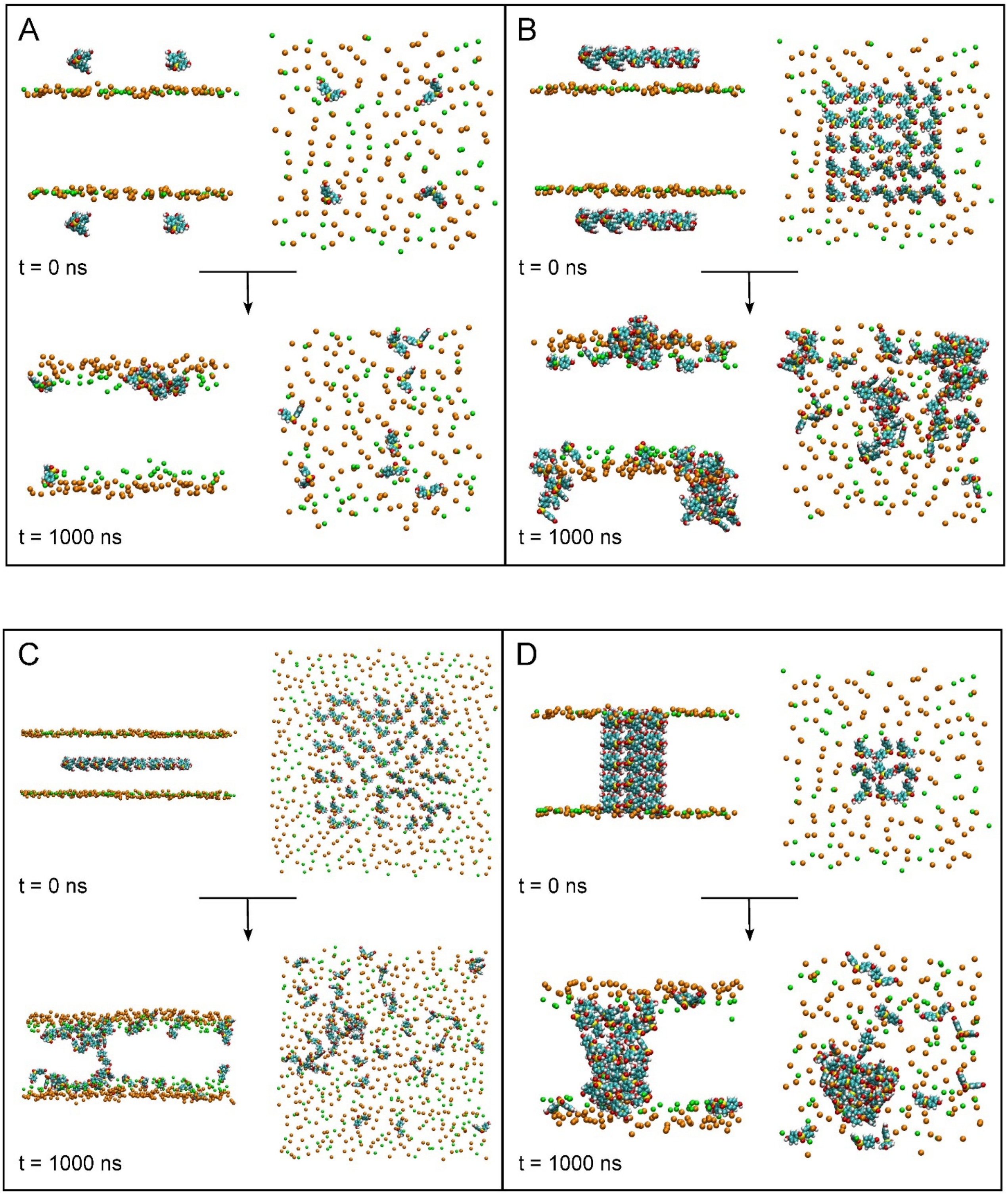

Figure S3. Lateral and apical views of the initial, t=0 ns, and final, t= 1000 ns, disposition of (A) system BPS_8, (B) system BPS_50 , (C) system BPS_44 , and (D) system BPS_45 (Table 1).The disposition of the BP molecules in each one of the systems are also displayed. The BP molecules are depicted in VDW representation, the phosphate atoms of the phospholipids, defining the upper and lower boundaries of the membrane, are depicted in VDW representation and orange colour and the oxygen atoms of cholesterol are depicted in VDW representation and green colour. The lipid and water molecules and the chloride and sodium ions have been removed for clarity.

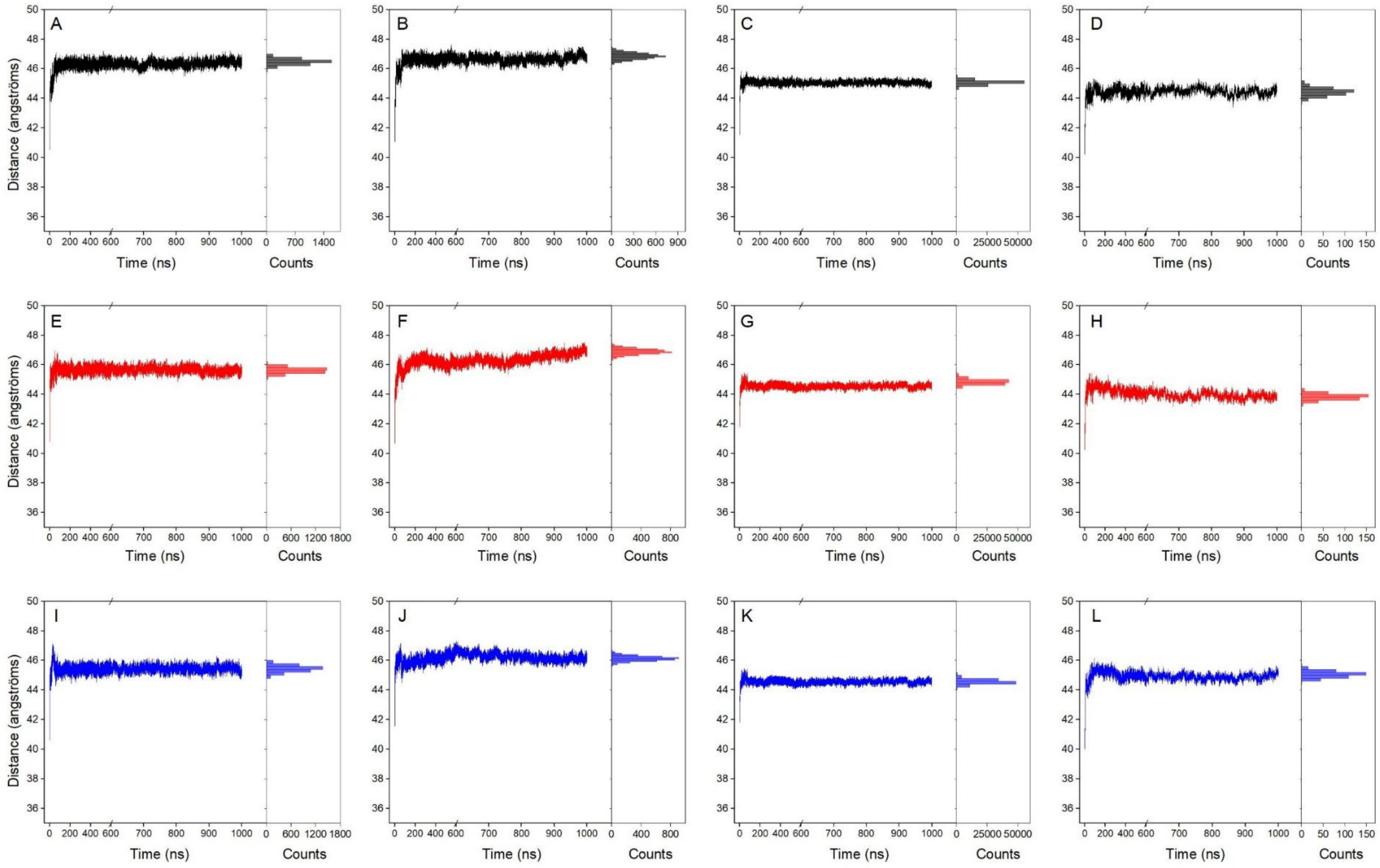

Figure S4. Time variation of membrane thickness for the whole simulation time and average histograms for the membrane thickness for the last 40 n of the simulation for (A) system BPA_8, (B) system BPA_50, (C) system BPA_44, (D) system BPA_45, (E) system BPF_8, (F) system BPF_50, (G) system BPF_44, (H) system BPF_45, (I) system BPS_8, (J) system BPS_50, (K) system BPS_44 and (L) system BPS_45.. Systems containing BPA are displayed in black colour, systems containing BPF in red colour and systems containing BP in blue colour. See text for details.

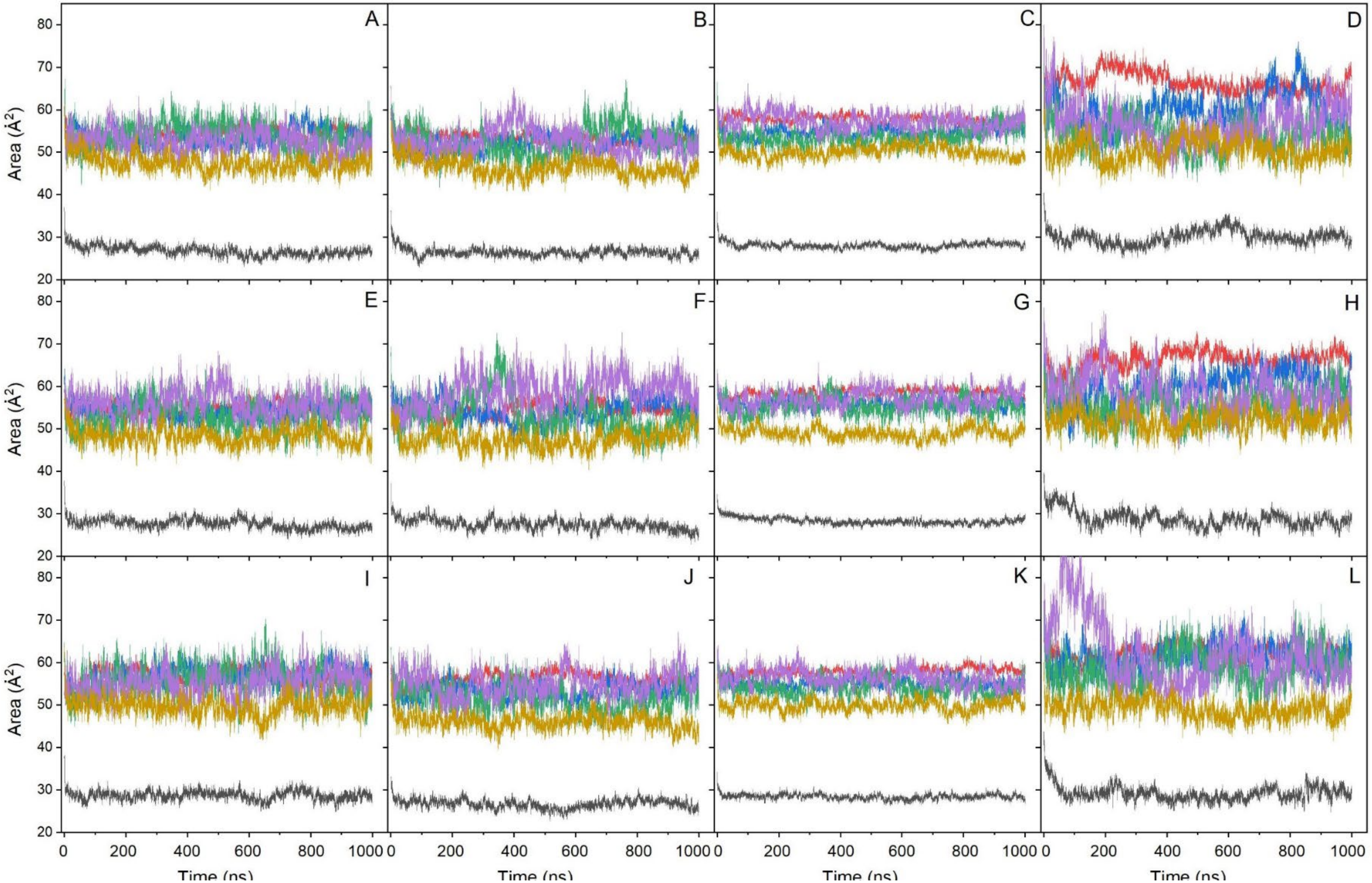

Figure S5. Time variation of lipid areas for the whole simulation time for the last 40 n of the simulation for (A) system BPA_8, (B) system BPA_50, (C) system BPA_44, (D) system BPA_45, (E) system BPF_8, (F) system BPF_50, (G) system BPF_44, (H) system BPF_45, (I) system BPS_8, (J) system BPS_50, (K) system BPS_44 and (L) system BPS_45.. Lipid areas correspond to POPC (red), POPE (blue), POPS (green), PI-3P (magenta), PSM (orange), and CHOL (black). See text for details.

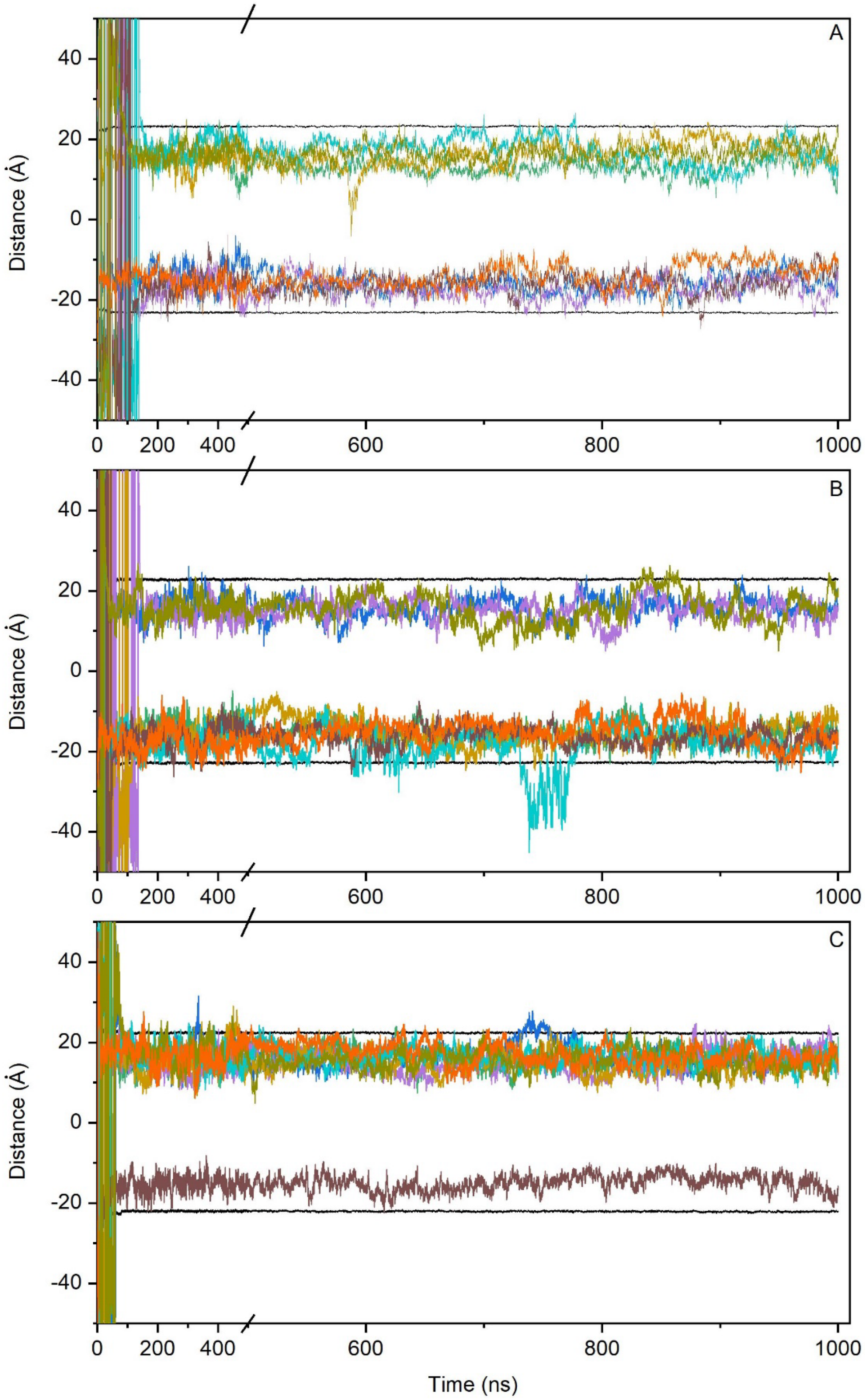

Figure S6. Time variation of the z-axis distance (middle of the membrane as a reference) for the different BP molecules in (A) system BPA_8 (8 molecules of BPA), (B) system BPF_8 (8 molecules of BPF) and (C) system BPS_8 (8 molecules of BP). The z-axis distance of the phosphate atoms of the phospholipids, defining the membrane surface, is depicted in black (upper and lower boundaries). See text for details.

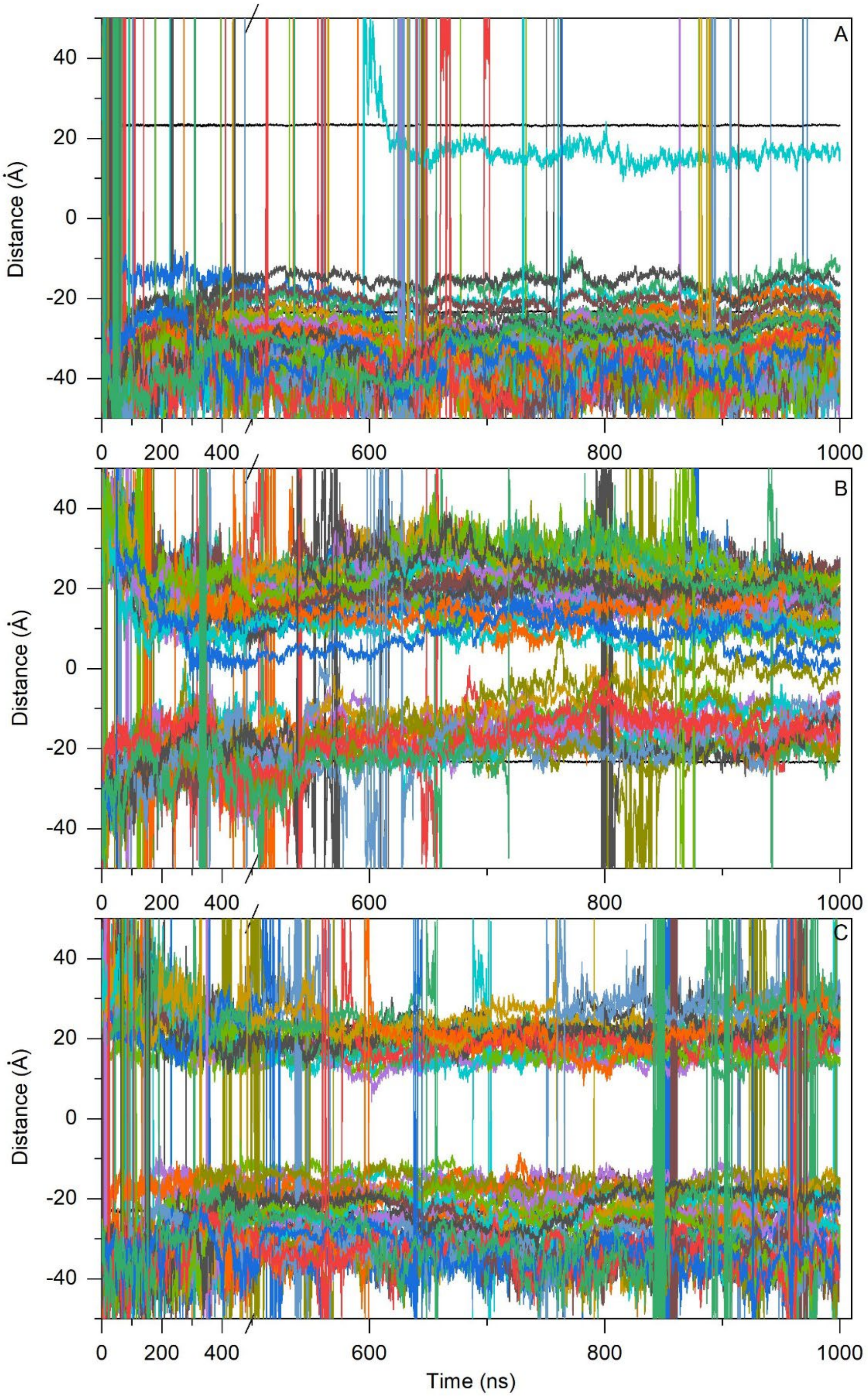

Figure S7. Time variation of the z-axis distance (middle of the membrane as a reference) for the different BP molecules in (A) system BPA_50 (50 molecules of BPA), (B) system BPF-50 (50 molecules of BPF) and (C) system BPS_50 (50 molecules of BP). The z-axis distance of the phosphate atoms of the phospholipids, defining the membrane surface, is depicted in black (upper and lower boundaries). See text for details.

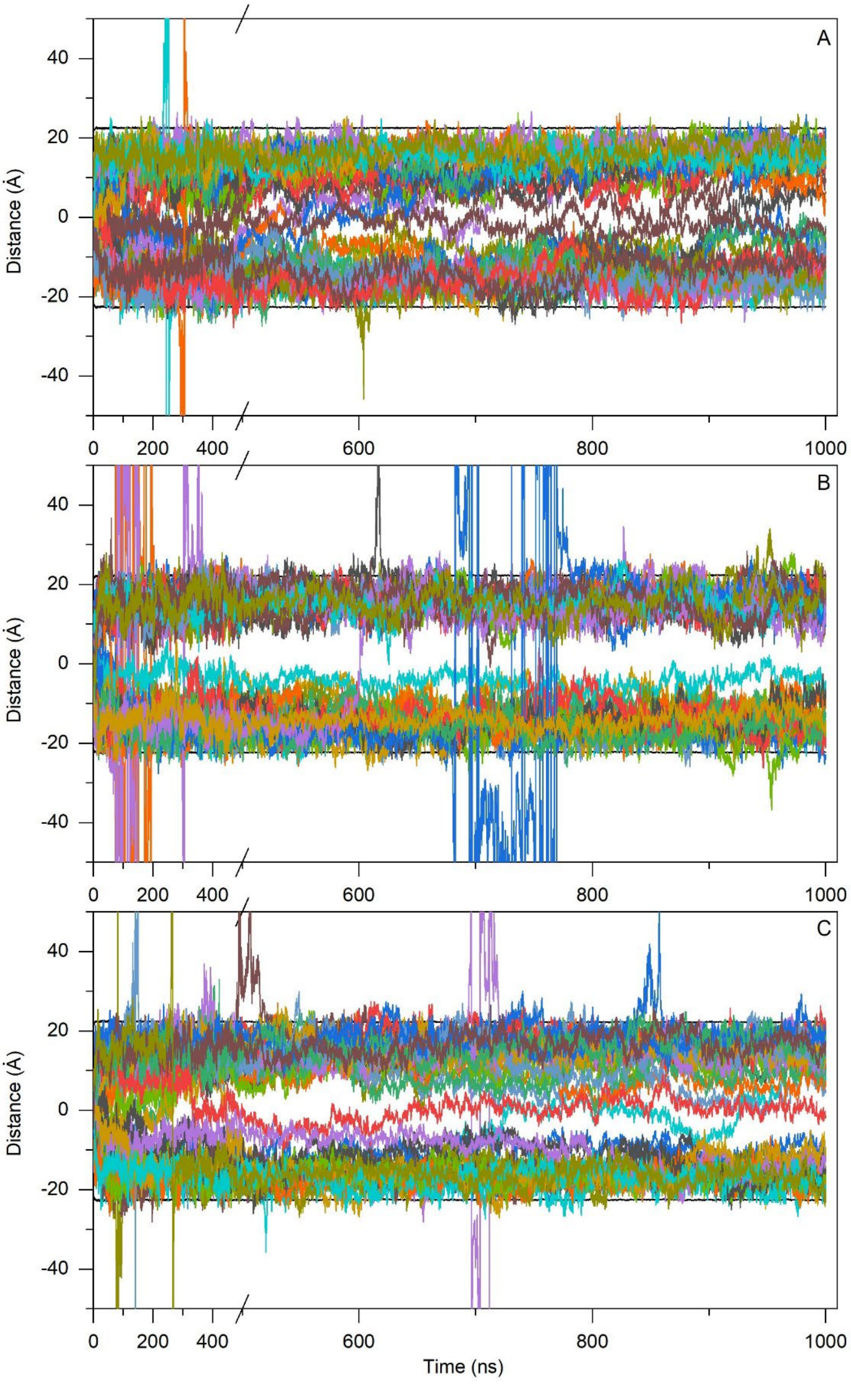

Figure S8. Time variation of the z-axis distance (middle of the membrane as a reference) for the different BP molecules in (A) system BPA_44 (44 molecules of BPA), (B) system BPF_44 (44 molecules of BPF) and (C) system BPS_44 (44 molecules of BP). The z-axis distance of the phosphate atoms of the phospholipids, defining the membrane surface, is depicted in black (upper and lower boundaries). See text for details.

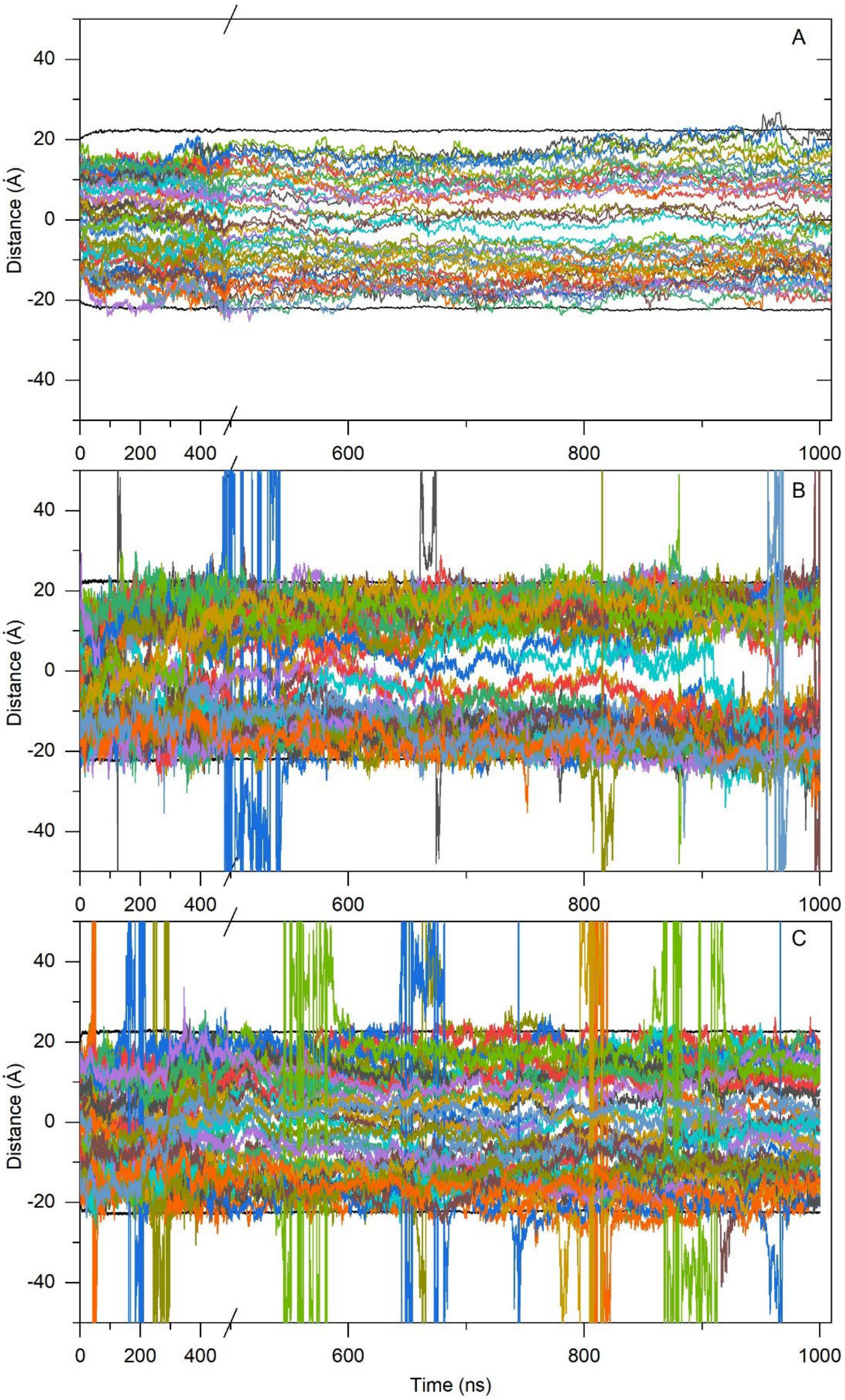

Figure S9. Time variation of the z-axis distance (middle of the membrane as a reference) for the different BP molecules in (A) system BPA_45 (45 molecules of BPA), (B) system BPF_45 (45 molecules of BPF) and (C) system BPS_45 (45 molecules of BP). The z-axis distance of the phosphate atoms of the phospholipids, defining the membrane surface, is depicted in black (upper and lower boundaries). See text for details.

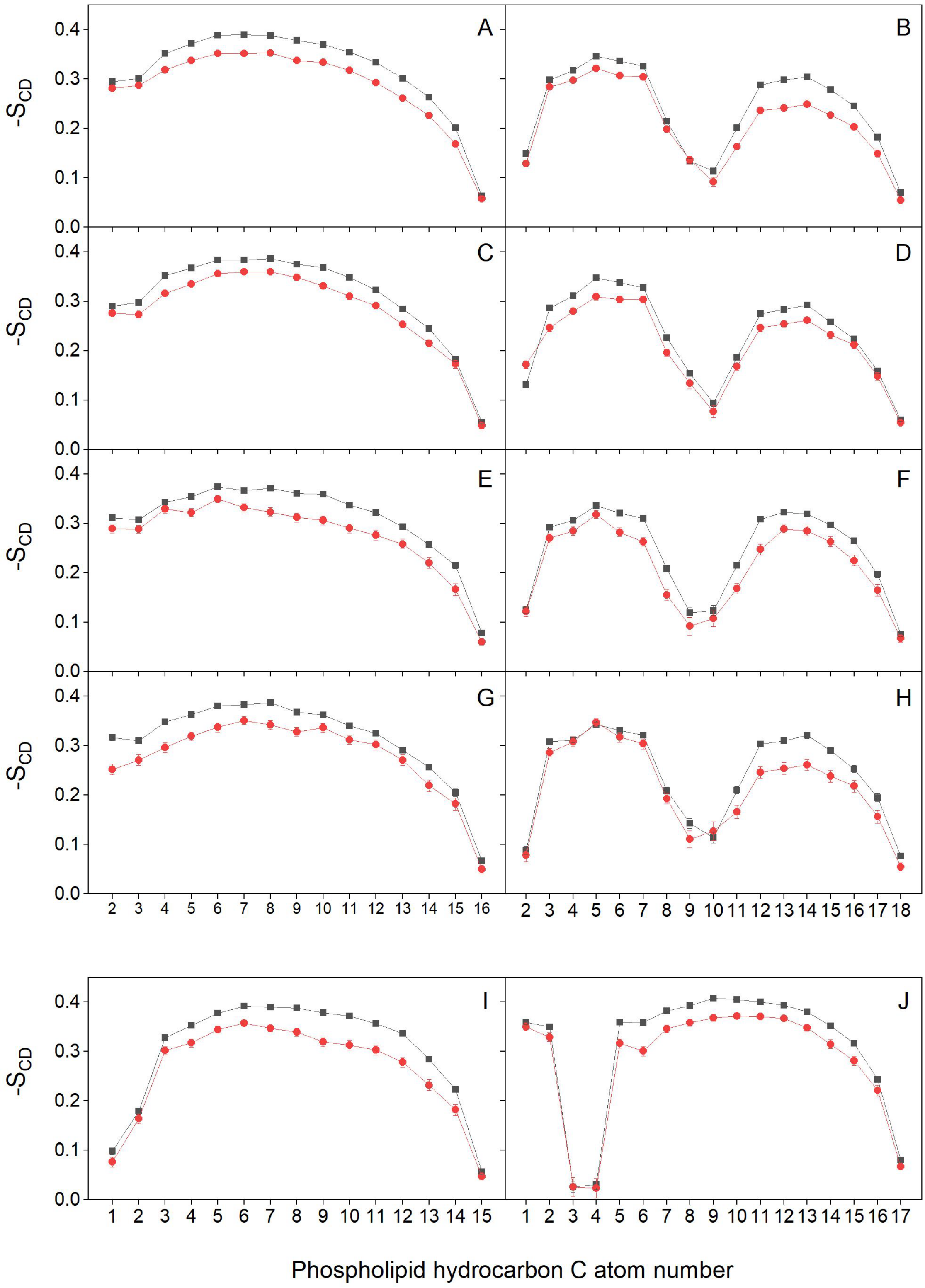

Figure S10. Average deuterium order parameter –S_{CD} calculated for the hydrocarbon chains of the phospholipids in system BPA_8. (A, C, E, G) oleoyl and (B, D, F, H) palmitoyl acyl chains of (A, B) POPC, (C, D) POPE, (E, F) POPS and (G,H) PI-3P as well as the palmitoyl (I) and sphyngosyl (J) acyl chains of PSM. The data correspond to the bulk phospholipid acyl chains (-■-) and the phospholipid acyl chains within 5 Å of BPA molecules (-●-). The analysis was carried out for the last 40 ns of simulation.

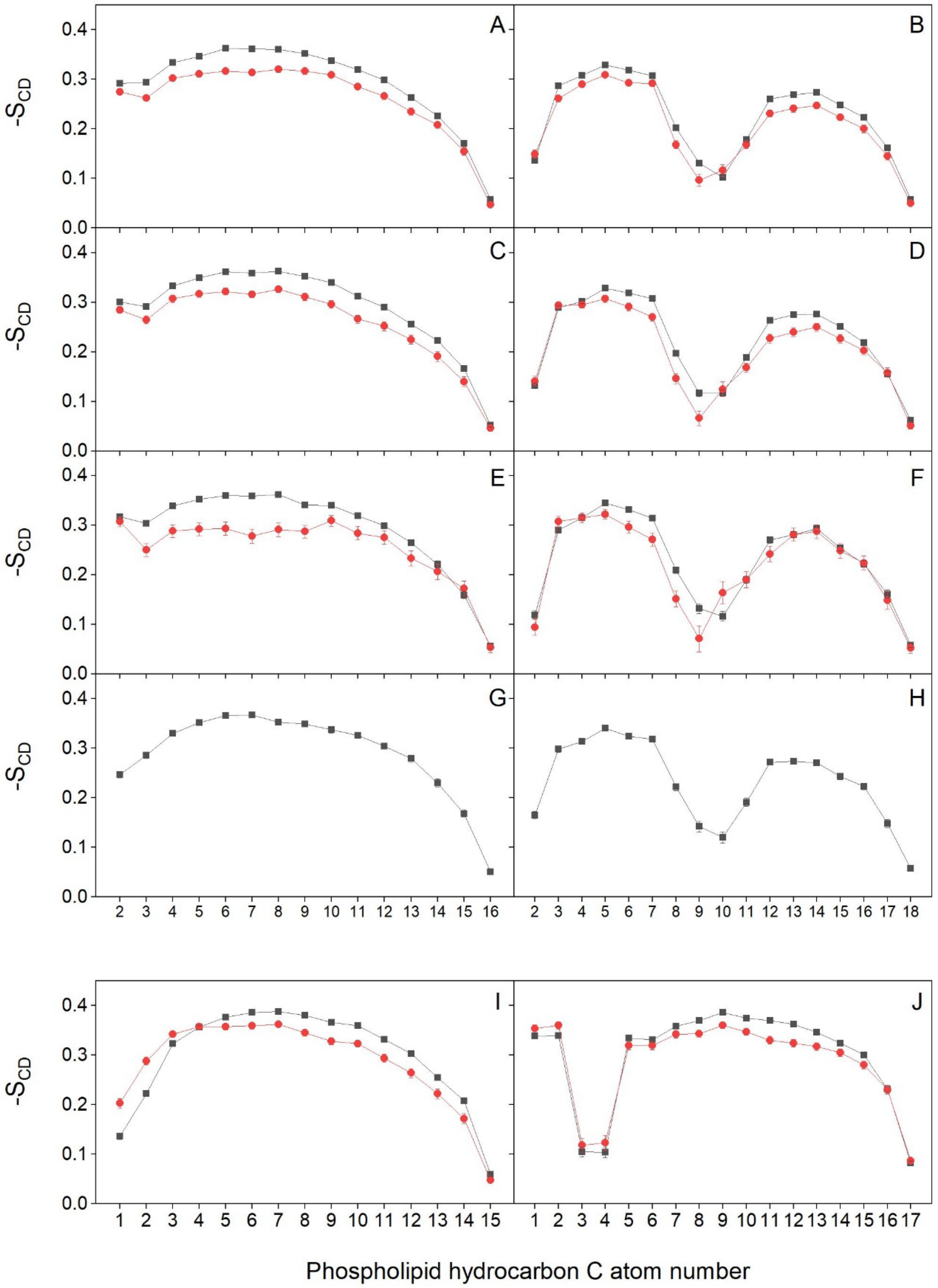

Figure S11. Average deuterium order parameter $-S_{CD}$ calculated for the hydrocarbon chains of the phospholipids in system BPF_8. (A, C, E, G) oleoyl and (B, D, F, H) palmitoyl acyl chains of (A, B) POPC, (C, D) POPE, (E, F) POPS and (G,H) PI-3P as well as the palmitoyl (I) and sphyngosyl (J) acyl chains of PSM. The data correspond to the bulk phospholipid acyl chains (-■-) and the phospholipid acyl chains within 5 Å of BPF molecules (-●-). The analysis was carried out for the last 40 ns of simulation.

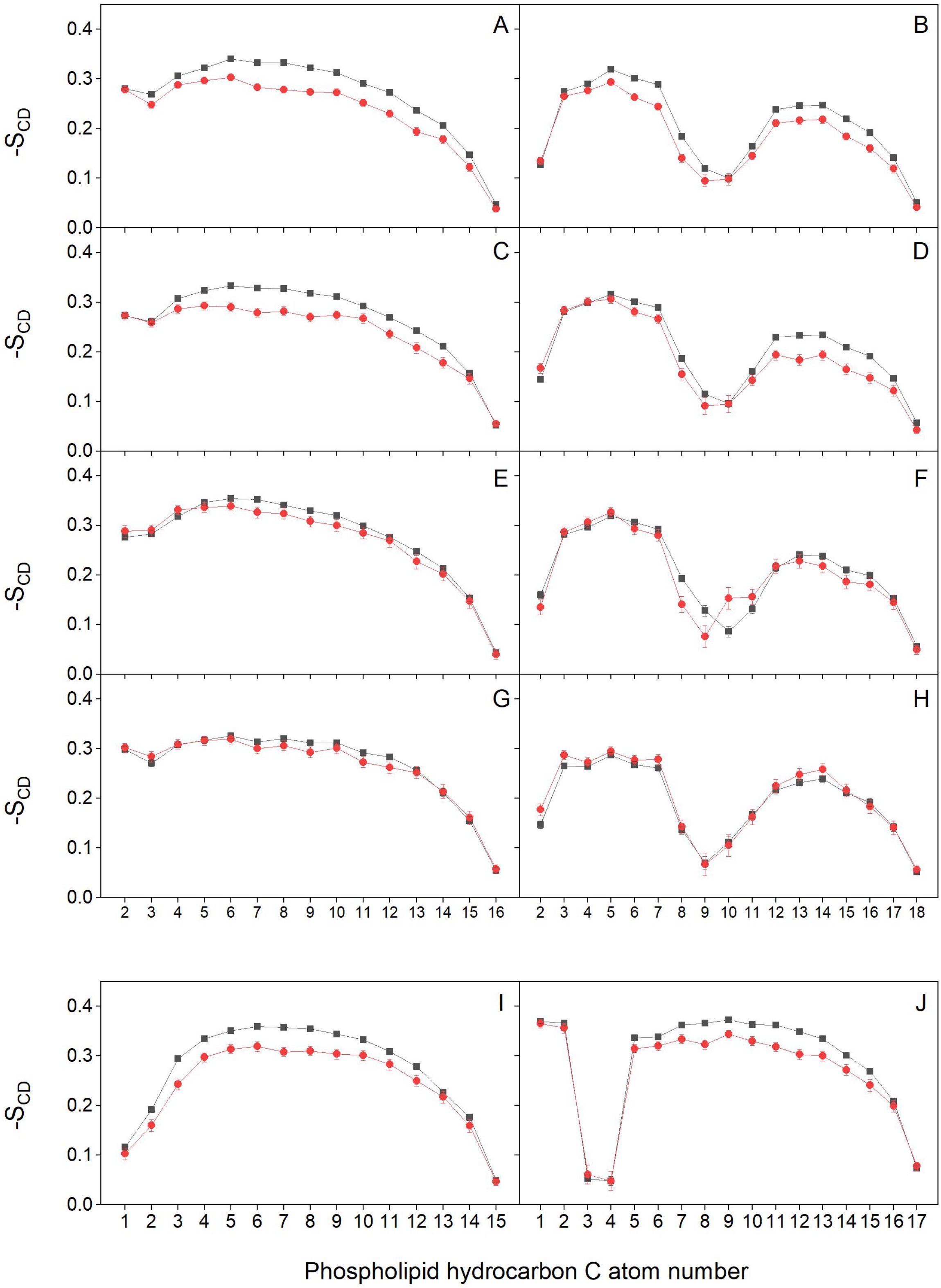

Figure S12. Average deuterium order parameter –S_{CD} calculated for the hydrocarbon chains of the phospholipids in system BPS_8. (A, C, E, G) oleoyl and (B, D, F, H) palmitoyl acyl chains of (A, B) POPC, (C, D) POPE, (E, F) POPS and (G,H) PI-3P as well as the palmitoyl (I) and sphyngosyl (J) acyl chains of PSM. The data correspond to the bulk phospholipid acyl chains (-■-) and the phospholipid acyl chains within 5 Å of BPS molecules (-●-). The analysis was carried out for the last 40 ns of simulation.

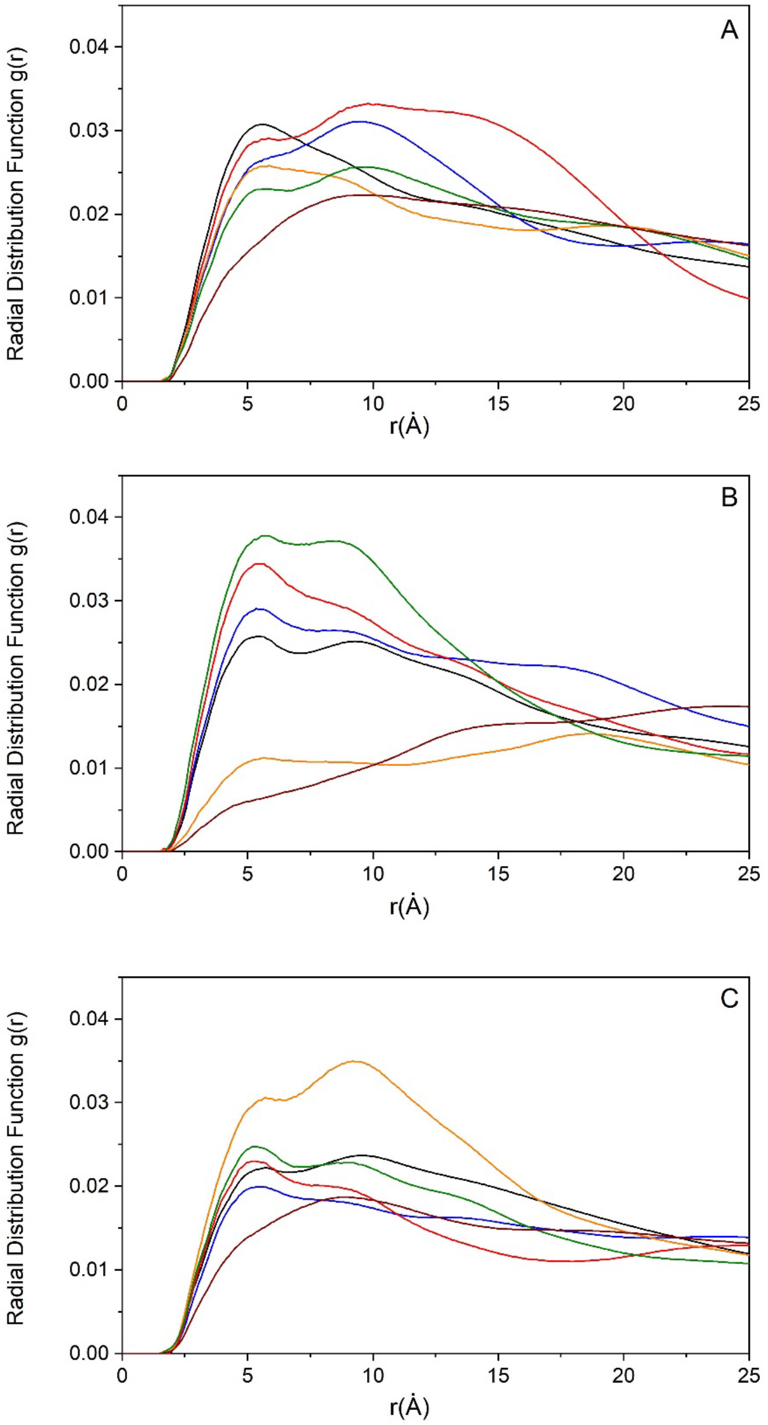

Figure S13. Radial distribution function, g(r), corresponding to the lipid molecules around the membrane-inserted bisphenol molecules for (A)) system BPA_8, (B) system BPF_8 and (C) system BPS_8. The lipids correspond to POPC (black), POPE (blue), PI-3P (orange), POPS (red), PSM (olive) and CHOL (wine). The analysis was carried out for the last 40 ns of simulation.

Table S1. Average molecular area (Å^2) and membrane thickness (Å) for the last 40 ns of the simulation for all the lipids in the systems studied in this work (mean ± SD).

		AREA (Å^2)						THICKNESS (Å)
		**POPC**	**POPE**	**POPS**	**PI-3P**	**PSM**	**CHOL**	
SYSTEMS								
**1**	**BPA_8**	53.9 ± 0.9	53.5 ± 1.3	54.4 ± 2.6	51.7 ± 1.9	47.8 ± 1.5	26.7 ± 0.6	46.5 ± 0.2
**2**	**BPA_50**	53.4 ± 0.9	53.9 ± 1.2	51.7 ± 1.5	51.1 ± 1.7	45.7 ± 1.5	25.8 ± 0.9	46.9 ± 0.2
**3**	**BPA_44**	56.9 ± 0.6	55.6 ± 0.8	55.8 ± 1.6	57.7 ± 1.4	48.7 ± 0.9	28.2 ± 0.6	45.1 ± 0.2
**4**	**BPA_45**	66.4 ± 2.4	54.4 ± 2.8	53.5 ± 2.8	60.4 ± 2.8	51.1 ± 2.1	29.8 ± 1.2	44.5 ± 0.3
**5**	**BPF_8**	54.7 ± 1.1	55.3 ± 1.2	54.1 ± 2.4	56.1 ± 2.1	47.6 ± 2.2	26.9 ± 0.6	45.6 ± 0.2
**6**	**BPF_50**	55.3 ± 0.8	54.5 ± 1.6	51.8 ± 2.1	57.5 ± 2.5	50.7 ± 1.8	25.5 ± 0.8	46.7 ± 0.3
**7**	**BPF_44**	57.6 ± 0.7	56.4 ± 0.8	54.7 ± 1.3	58.1 ± 1.5	49.5 ± 1.1	28.5 ± 0.5	44.6 ± 0.2
**8**	**BPF_45**	67.2 ± 1.6	59.8 ± 3.6	58.5 ± 3.4	55.0 ± 2.7	53.4 ± 2.2	28.2 ± 1.0	43.9 ± 0.2
**9**	**BPS_8**	57.3 ± 1.0	57.5 ± 1.7	51.9 ± 3.2	54.9 ± 2.4	50.3 ± 2.5	28.7 ± 0.8	45.4 ± 0.2
**10**	**BPS_50**	55.5 ± 0.9	56.1 ± 1.2	52.4 ± 2.1	55.2 ± 2.1	44.4 ± 1.5	25.9 ± 0.8	46.1 ± 0.2
**11**	**BPS_44**	57.8 ± 0.6	54.7 ± 1.2	54.5 ± 1.9	54.7 ± 1.5	50.1 ± 1.4	28.5 ± 0.6	44.8 ± 0.2
**12**	**BPS_45**	61.1 ± 1.2	61.2 ± 2.2	59.8 ± 2.5	57.3 ± 2.1	49.3 ± 1.8	29.7 ± 1.2	45.1 ± 0.2
